# Plant protein-derived anti-breast cancer peptides: sources, therapeutic approaches, mechanisms, and nanoparticle design

**DOI:** 10.3389/fphar.2024.1468977

**Published:** 2025-01-17

**Authors:** Deju Zhang, Ying Yuan, Qingdong Zeng, Juan Xiong, Yiming Gan, Kai Jiang, Ni Xie

**Affiliations:** ^1^ Biobank, Shenzhen Second People’s Hospital, First Affiliated Hospital of Shenzhen University, Shenzhen, China; ^2^ Guangdong Key Laboratory for Biomedical Measurements and Ultrasound Imaging, National-Regional Key Technology Engineering Laboratory for Medical Ultrasound, School of Biomedical Engineering, Shenzhen University Medical School, Shenzhen, China; ^3^ Food and Nutritional Sciences, School of Biological Sciences, The University of Hong Kong, Pokfulam, Hong Kong SAR, China; ^4^ Hengyang Medical School, University of South China, Hengyang, China; ^5^ Plant Science, School of Biological Sciences, The University of Hong Kong, Pokfulam, Hong Kong SAR, China; ^6^ Eastern Institute for Advanced Study, Eastern Institute of Technology, Ningbo, China; ^7^ Department of Thermal Science and Energy Engineering, University of Science and Technology of China, Hefei, China

**Keywords:** plant-derived peptides, breast cancer, nanoparticles, anti-breast cancer, legume protein

## Abstract

Breast cancer causes the deaths of approximately 685,000 women annually, posing a severe threat to women’s health. Consequently, there is an urgent need for low-cost, low-toxicity and effective therapeutic methods to prevent or mitigate breast cancer progression. PDBP are natural, non-toxic, and affordable substances and have demonstrated excellent anti-breast cancer activities in inhibiting proliferation, migration, and invasion, and promoting apoptosis both *in vitro* and *in vivo*, thus effectively preventing or inhibiting breast cancer. However, there are no comprehensive reviews summarizing the effects and mechanisms of PDBP on the treatment of breast cancer. Therefore, this review described the inhibitory effects and mechanisms of active peptides from different plant protein sources on breast cancer. Additionally, we summarized the advantages and preparation methods of plant protein-derived anticancer peptide-encapsulated nanoparticles and their effects in inhibiting breast cancer. This review provides a scientific basis for understanding the anti-breast cancer mechanisms of PDBP and offers guidance for the development of therapeutic adjuvants enriched with these peptides.

## 1 Introduction

Breast cancer is the most prevalent type of cancer among female patients and is also the leading cause of cancer-related deaths (Wang and Wu, 2023). According to the data reported by the World Health Organization, approximately 2.26 million new cases of breast cancer were diagnosed in 2020, comprising 1/8 cancer patients globally, making breast cancer one of the most aggressive and widespread cancers in the world (Lv et al., 2023). Conventionally, the treatments for breast cancer include chemotherapy, radiation, surgery, and immunotherapy, but these methods remain inadequate and unsatisfactory with respect to outcomes and prognosis (Wang and Wu, 2023). For instance, surgical methods not only compromise women’s body image but also have a 25%–60% chance of resulting in chronic pain, while radiation therapy and chemotherapy have been demonstrated to adversely influence the healthy organs of patients (Nwachukwu and Aluko, 2019). Therefore, it is necessary to develop new effective cancer treatment methods, especially those with minimal side effects, high efficacy, low cost, and high patient compliance, in order to maximize the cure rate and improve the life quality of breast cancer patients. According to research by Anand et al. (2008), only 5%–10% of diagnosed cancer cases are attributed to genetic defects, while the remaining cases of cancer are attributed to factors such as environment, diet, and lifestyle. Additionally, epidemiological evidence suggests that changing dietary nutrient intake ratios and food consumption patterns can reduce the occurrence of various types of cancers (Hernández-Ledesma and Hsieh, 2017). Therefore, consuming anti-cancer foods or isolating active ingredients from food for the treatment of breast cancer has drawn the attention of scholars.

**FIGURE 1:**
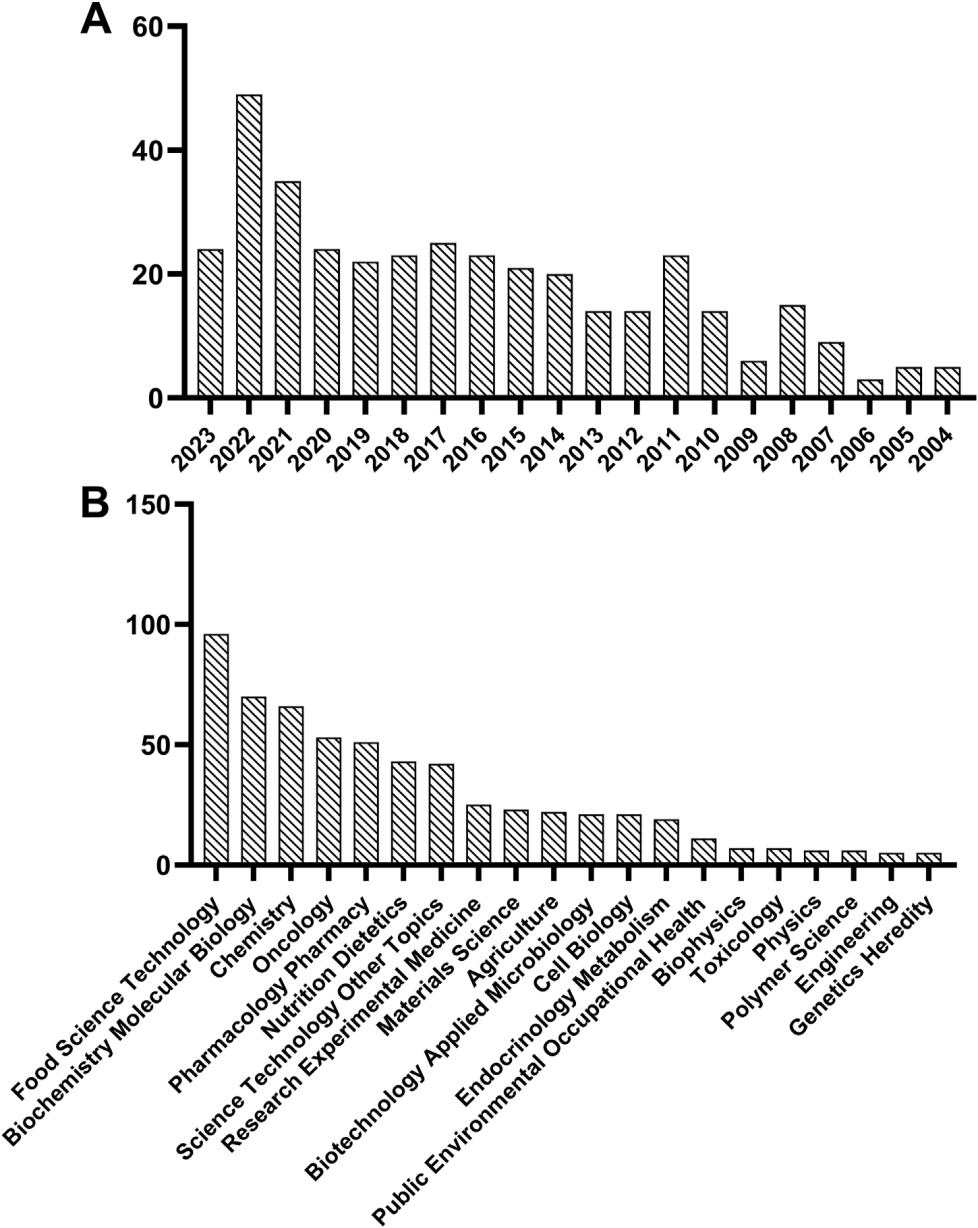
Statistics on food peptide and breast cancer in web of science based on publication year **(A)** and research direction **(B)**.

Bioactive peptides are kinds of specific protein fragments consisting of approximately 2–20 amino acid residues and typically have a beneficial influence or have a physiological impact on the life activities of living organisms (Jia et al., 2021). Over the past 2 decades, scientists have identified a number of bioactive peptides from various organisms and have conducted extensive research on their biological activities, such as antimicrobial, antioxidant, antithrombotic, antidiabetic, and anticancer activities (Du et al., 2019; Chai et al., 2020). Food-derived bioactive peptides are primarily derived from animal, plant, and microbial proteins (Jia et al., 2021). Their fragments are inactive within the parent protein but could be extensively released by fermentation, enzymatic hydrolysis, or other processing methods (Liu et al., 2020). As shown in Figure 1A, research on breast cancer and food peptides has been on the rise over the past 2 decades, as retrieved by Web of Science. Besides, the category of “Food Science Technology” occupies a dominant role in the study of food peptides for breast cancer treatment (Figure 1B). Plant-based bioactive peptides mainly come from proteins such as soy (Hsieh et al., 2023), chickpea (Xue et al., 2015), black soybeans (Ren et al., 2021), rice (Zhang L. Y. et al., 2023), walnut (Wang J. P. et al., 2022), corn (Wei et al., 2022), barley (Tok et al., 2021), spirulina (Suo et al., 2022), seaweed (Cian et al., 2022), etc. Compared to animal-derived and microbial-derived bioactive peptides, plant-derived peptides offer a rich variety, lower cost, reduced allergenicity, higher safety, and greater sustainability (Zhu et al., 2023), all of which has led to their growing popularity in the food and nutritional supplement markets and among certain consumers (Fan H. X. et al., 2022).

Due to the remarkable advantages of PDBP, scholars have explored the application of these peptides in the treatment or prevention of breast cancer. Hsieh et al. (2023) reported that seed-derived peptide lunasin reduced the cell viability of breast cancer cells (MDA-MB-231 and MCF-7 cell), while the growth of normal human breast epithelial cells (MCF-10A) was not affected. He attributed the above results to the fact that lunasin modulated the accumulation of the inflammatory factor Interleukin-6 (IL-6), inhibited the secretion of vascular endothelial growth factor and reduced the expression of leptin receptor and estrogen receptor α. Similarly, amaranth seed protein-derived peptides have also been demonstrated to inhibit the cell growth of triple-negative breast cancer cells to 50% at a concentration of 48.3 ± 0.2 μg/mL, which was possible because the amaranth seed peptides could induce DNA fragmentation, membrane integrity loss, as well as increase caspase 3 activity (Taniya et al., 2020). Although the effects and mechanisms of PDBP to inhibit breast cancer have been extensively explored, reviews summarizing the progress in this field are still lacking.

To clarify the great promise of plant protein active peptides for the treatment of breast cancer, we conducted the following work. First, we offered an overview regarding the sources, sequences and anti-breast cancer effects of different PDBP and revealed the underlying mechanisms by which they exhibit anti-breast cancer effects. Second, this review also summarized the advantages and preparation methods of plant protein-derived peptides-encapsulated nanoparticles and their therapeutic effects on breast cancer. Our study will be of great interest to the deep processing of plant proteins and the clinical application of PDBP.

## 2 Methods

The following electronic databases were searched from the beginning to October 2024: PubMed, Web of Science, Google Scholar, and CNKI. The keywords of plant peptides and/or breast cancer were used truncated with other relevant topic terms, such as soybean peptides, lunasin, chickpea peptides, pea peptides, migration, invasion, cell viability, anti-breast cancer, apoptosis, action mechanism, nanoparticles, p53, mitochondrial apoptotic pathway, and PI3K-Akt. In addition, we also found some literature from references as a supplement.

## 3 Plant protein-derived anti-breast cancer peptides

**FIGURE 2:**
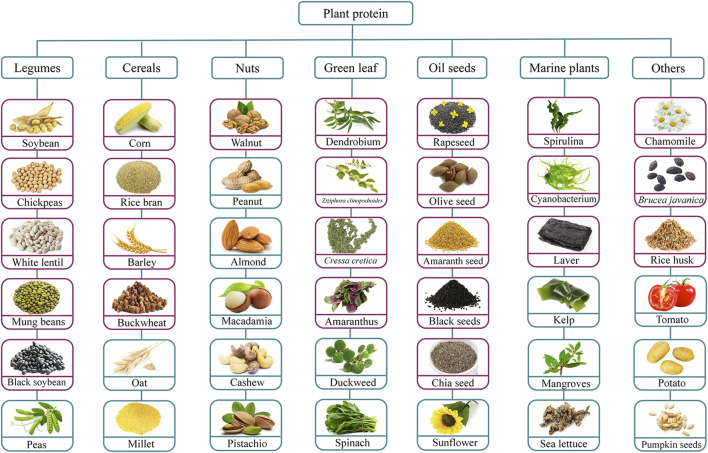
Anti-breast cancer peptides derived from different plant sources. Figure is adapted from Zhang et al. (2024a). The red purple box indicates that studies have been done to extract anti-breast cancer peptides from this plant protein, and the blue box indicates that no studies have been done to extract anti-breast cancer peptides from this plant protein.

Plant proteins not only meet 70% of the world’s protein needs but also serve as an important way of obtaining non-essential amino acids at low cost, thus attracting increasing attention in commercial applications and daily diets (Görgüç et al., 2020). Typically, PDBP are enclosed within plant proteins and can be rapidly released after digestion by gastrointestinal proteases, after which they are absorbed by intestinal epithelial cells and enter the bloodstream to exert their biological effects. As indicated in Figure 2, legumes, grains, marine plants, and oilseed crops are all excellent sources of PDBP (Görgüç et al., 2020).

### 3.1 Legume protein-derived anti-breast cancer peptides

#### 3.1.1 Characteristics of legume proteins

Legumes are of crucial importance in the traditional diets of many countries throughout the world and they could provide a rich amount of protein, minerals (iron, zinc, calcium), and vitamins (thiamine, niacin, biotin, riboflavin, folate) (Da Silva et al., 2018; Kumar and Pandey, 2020). The protein content in legumes is higher than that in grains and oil crops, making them an excellent choice for preparing bioactive peptides and hydrolysis products for functional and nutritional foods (Hernandez et al., 2024; Zhang et al., 2024b).

Most legumes have a 20%–30% protein content, while soybeans and lupins contain about 40% protein (Table 1). Legume proteins are categorized into several types, including protease inhibitors, amylase inhibitors, lectins, and storage proteins, with storage proteins being predominant in legume seeds (Klupšaitė and Juodeikienė, 2015). According to Tan et al. (2023), storage proteins are divided into four classes: globulins (soluble in salt solutions), albumins (soluble in water), glutelins (soluble in acid or alkali), and prolamins (soluble in alcohol). Among these, globulins dominate in legume seeds, with legumin, vicilin, and convicilin being the three main types of legume globulins (Carbonaro and Nucara, 2022).

**TABLE 1:** Protein content of plants commonly used to extract anti-breast cancer peptides.

Classification	Plants	Crude protein (w/w, %)	Composition	References
Legumes	Soybean	40%–41%	90% globulins (mainly consisting of 7S (β conglycinin) and 11S (glycinin)) and 10% albumins	Jung et al. (2003), Zhang et al. (2024a)
	Chickpea	16.1%–26.7%	8%–12% albumins, 53%–60% globulins, 3%–7% prolamins, and 19%–25% glutelins	Grasso et al. (2022), Zhang et al. (2024a)
	Mung beans	24%	60% globulins, 25% albumins, and other globulins including basic-type 7S and legumin-type11S	Nair et al. (2013), Zhang et al. (2024a)
	Pea	20%–25%	Legumin (11S), vicilin (7S)	Shanthakumar et al. (2022), Zhang et al. (2024a)
	Black soybean	32.1%–43.89%	Globulin (glycinin (11S) and β-conglycinin (7S))	Kumar et al. (2023)
	Lupin	About 40%	25% albumins and 75% globulins	Lo et al. (2021), Zhang et al. (2024a)
	Hemp	20%–25%	20%–40% albumin and 60%–80% globulin (mainly 7S)	Wang and Xiong (2019), Burton et al. (2022)
	Cowpea	20.3%–39.4%	globulins (vicilins or 7S globulins), albumins, glutelins, and prolamins	Gonçalves et al. (2016)
	Kidney bean	20%–25%	50%–81% globulins (11S), 40%–50% globulins (7S), phaseolin	Punia et al. (2020), Gravel and Doyen (2023)
	Moth bean	23%–26%	4.5%–26% albumins, 10%–57% globulins, glutelins and prolamins	Dhull et al. (2024)
	Lentil	20.6%–31.4%	16% albumins, 70% globulins, 11% glutelins and 3% prolamins	Jarpa-Parra (2018)
	Peanut	22%–30%	35%–95% 7 S globulin, 50% 11 S globulin, 13%–38% 2 S albumin, Profilin	Toomer (2018)
Cereals	Corn	6%–12%	Zein, albumin and globulin	Ai and Jane (2016)
	Oat	15%–20%	70%–80% globulin, 4%–14% prolamin, 1%–12% albumin, and less than 10% glutelin	Spaen and Silva (2021)
	Rice	6.3%–15%	75%–81% glutelin, 7%–17% globulin, 5%–10% albumin and 3%–6% prolamin	Amagliani et al. (2017)
	Wheat	12.35%–16.92%	6%–10% albumins, 5%–8% globulins, 35%–40% prolamins, and 40% glutelins	Ye et al. (2018), Zhang et al. (2024a)
	Potato	1.76%–2.95%	40%–60% patatins, 20%–30% protease inhibitors, and other high-molecular weight proteins	de Haan et al. (2019), Zhang et al. (2024a)
	Buckwheat	About 12%	Albumin, globulin, prolamin, and glutelin	Zhu, 2021; Zhang et al. (2024a)
	Barley	8%–30%	30%–50% hordeins, 35%–45% glutelins, and protein Z	Jaeger et al. (2021)
	Rye	8%–15%	29%–40% albumins, 8%–11% globulins, 17%–19% prolamins and 9%–15% glutelins	Németh and Tömösközi (2021)
	Millet	7%–12%	8% albumins, 4% globulins, 41% prolamins, and 41% glutelins	Bangar et al. (2022)
Oil crop	Rapeseed	17%–26%	60%–65% of globulins and 30%–35% of albumins	Chmielewska et al. (2021)
	Walnut	18%–24%	Glutenin, gliadin, globulin, and albumin	Wen et al. (2023)
	Olive	1.6%	Albumins, globulins, prolamins, and glutelin	Ghanbari et al. (2012), Rahman et al. (2024)
	Sunflower seed	20.78%	55%–60% globulins, 17%–23% albumins, 11%–17% glutelins, and 1%–4% prolamins	Anjum et al. (2012), Zhang et al. (2024a)
	Sesame	25%	47.42% glutelin, 22.84% albumin, 10.52% globulin, and 1.29% prolamin	Johnson et al. (1979), Zhang et al. (2024a)
	Flaxseed	20%–30% protein	15%–30% albumin, 70%–85% globulin, prolamin, glutelin	Bekhit et al. (2018), Peng et al. (2022)
	Coconut powder	33%	61.9% globulin, 30.6% albumin, 4.1% glutelin, and 1.1% prolamins	Naik et al. (2012), Zhang et al. (2024a)
	Chia seed	26%	Prolamins, glutelins, albumins, and globulins	Grancieri et al. (2019), Quintal-Bojórquez et al. (2021)
	Almond	10%–29%	40.5% albumin, 18.3% globulin A, 30.5% globulin B, 1.0% prolamin, and 1.0% glutelin	Massantini and Frangipane (2022), Zhang et al. (2024a)
Marine plant	Spirulina platensis	60%–70%	a and b protein subunits of 17,000 and 19,500 Da	Saranraj and Sivasakthi (2014)
	Cyanobacteria	17.9%–71%	Allophycocyanin, phycocyanin, phycoerythrin, phycoerythrocyanin	Pagels et al. (2019), Grossmann et al. (2020)
	Porphyra yezoensis	28%–39%	Allophycocyanin, phycocyanin, phycoerythrin, phycoerythrocyanin	Cao et al. (2016), Grossmann et al. (2020)
Others	Amaranthus cruentus	14%	48.9%–65% albumins, 13.7%–18.1% globulins, 1.0%–3.2% prolamins, and 22.4%–42.3% glutelins	Wolosik and Markowska (2019)
	Pumpkin seed	21.31%	2S albumin, 11S globulin, vicilin, oleosin	Dotto and Chacha (2020), Agrawal and Shahani (2021)
	Nigella sativa	26.7%		Khurshid et al. (2020)

As demonstrated in Table 1, common types of leguminous plants include soybean, peanut, common bean, chickpea, lentil, lupin, fava bean, and pea (Juárez-Chairez et al., 2022). Additionally, some novel legume proteins such as kidney beans, cowpeas, and black soybeans are gradually being investigated as rich sources of anticancer peptides (Fang et al., 2010; Luna-Vital et al., 2016; Avilés-Gaxiola et al., 2020; da Silva et al., 2021). Due to the presence of globulins and albumins, legume proteins contain abundant aspartic acid, glutamic acid, lysine, and sulfur-containing amino acids, which may contribute to the high anti-colorectal, anti-breast, anti-prostate, anti-stomach, and anti-pancreatic cancer activities of legume-derived peptides (Cellarier et al., 2003; Yamaguchi et al., 2016; Chiangjong et al., 2020; Carbonaro and Nucara, 2022).

#### 3.1.2 Anti-breast cancer activities of legume protein-derived peptides

Peptides that may inhibit breast cancer have been identified in several legume proteins, involving soybean (Hao et al., 2022), chickpea (ANDISFNFVRFNETNLILGG, RQSHFANAQP) (Xue et al., 2015; Grasso et al., 2022), lentil (EVASYSGW, FFADTGIK) (Kuerban et al., 2020), black soybean (DFPLDNEHHNMLENGG) (Fang et al., 2010), etc (Table 2). These plant peptides can inhibit the proliferation, adhesion, and migration, promote apoptosis, induce cell cycle G0/G1 phase arrest of breast cancer cells, as well as lead to overexpression of apoptosis-related proteins such as caspase 3, caspase-7, caspase-8, thereby hindering the progression of breast cancer.

**TABLE 2:** Inhibitory effect and mechanism of plant protein-derived active peptides on breast cancer progression.

Protein source	Processing method	Peptides	Model	Anti-breast cancer effects	Pathway	References
Soybean	Alcalase (pH 7.0, 55°C, 45 min)	Peptide fractions: <5, 5–10 and 10–50 kDa	MCF-7 cells were treated with 200, 400, 600, 8000 and 1000 μg/mL of peptide fractions (5–10 kDa)	IC_50_: 608–654 μg/mL Cell inhibition: 63%	Anti-proliferation	Rayaprolu et al. (2017)
Soybean	Pepsin and trypsin hydrolysis	Peptide fractions:10 kDa	MCF7 and MDA-MB-231 cells were treated with 1 and 10 mg/mL of peptide fractions	IC_50_: 19.99 mg/mL Apoptotic cells: 63% after 12 h treatment		Marcela et al. (2016)
Soybean	Fermented by *Lactobacillus acidophilus*, *Lactobacillus bulgaricus*, *Streptococcus lactis*, *Bifidobacteria*, and yeasts	Peptide fractions: <3, 3–10 and 10–50 kDa	Breast cancer cells and mice were treated with 1, 10, and 20 mL/kg fermented soybean	Cell viability: MCF-7, BT474, MDA-MB-453, and SK-BR-3↓ Tumor weight↓ GOT, GPT, Creatinine↑	Trigger ROS and apoptosis	Chang et al. (2002)
Ground beans	FPLC purification and SDS-PAGE	KTCENLADTY	MCF-7 cells were treated with 0.05, 0.1, 0.15, 0.2, 0.25, 0.3, 0.35, 0.4, 0.45 and 0.5 mg/mL peptides	Cell viability↓		Wong and Ng (2005)
Chickpea	Enzymatic hydrolysis with 2% alcalase (pH 8) for 1 h at 50°C	Peptide mixtures	MCF-7 and MDA-MB-231 cells were treated with peptide mixtures	IC_50_: 0.60–0.63 mg/mL		Wong and Ng (2005)
Chickpea	Purification and SDS-PAGE	ANDISFNFVRFNETNLILGG	MCF-7 cells were treated with 0, 60, 200 and 600 nM peptides	IC_50_: 0.2 μM Apoptosis, membrane depolarisation↑ G2/M phase arrest rosed from 25.3% to 44.7% Fas, caspase-8, Truncated BID, p53, truncated lamin A/C↑	Causes an arrest in the G2/M phase, death receptor-mediated pathway↑	Lam and Ng (2011)
Chickpea	Chemical synthesis	RQSHFANAQP	MCF-7 and MDA-MB-231 cells were treated with 0.5, 1.0, 1.5, 2.0, and 2.5 µM peptides	EC_50_: 2.38 µM for MCF-7 and 1.50 µM for MDA-MB-231 ROS levels↓ p53↑	p53 was combined with peptide via hydrogen-bonds	Xue et al. (2015)
Mung bean	Enzymatic hydrolysis with alcalase (pH 8) for 3.5 h at 37°C	Small molecular weight peptide mixtures	MCF-7 and MDA-MB-231 were treated with 10, 25, 50, 75 and 100 mg/mL of peptide mixtures	IC_50_: 0.32–0.73 mg/mL for MCF-7 and 0.26–0.54 mg/mL for MDA-MB-231		Gupta et al. (2018)
Mung bean	*Rhizopus* sp. Strain 5351, 48h, 30°C	Peptide mixtures	Balb/c mice injected with 4T1 cells were fed with 200 mg/kg or 1000 mg/kg mung bean peptide extracts	Tumor volume↓ CD4 and CD8 T cells↑ IL-2 and IFN-γ↑ SOD, NO↓, MDA↑		Yeap et al. (2013)
Moth bean seed	Alcalase was used at a ratio of 1:100 for 3 h at 65°C	30–50, 10–30, 5–10, 3–5 and 1–3 kDa	MCF-7 cells were treated with 0–200 μg/mL peptide fractions	IC_50_: 46.93 μg/mL; oxidative DNA damage↓		Bhadkaria et al. (2023)
Lentil	Trypsin was used at a ratio of 1:20 for 12 h at 37°C	EVASYSGW, FFADTGIK, TSFFDPAGG, LPAKSSAPK, LHVGDTEK, ESTYGILD, VYKIEDM, LECTSCDK, DDHDLKR, RNGIIK, LAVNPKSLG, YILNYVN, PNIHYVR, VHVSLK, HNEQLEK, QVIDGIPN, QLQVLAGGL, EKLAVNPK, PLIRSLAK, KSISTFSK, TQTFGNET, NHGMHFR, NQLLVNR, VACYMGR, STWK, KEAAHK, SCSAR	MCF-7 cells were added with 5, 10, 15 and 20 mg/mL peptide fractions (≤3 kDa)	IC_50_: 12.27 mg/mL		Kuerban et al. (2020)
Black soybean	Purification using Superdex 75 10/300 GL column	DFPLDNEHHNMLENGG	MCF-7 cells were treated with 20, 40, 60, 80 and 100 µM peptide fractions	Cell viability↓		Fang et al. (2010)
Lunasin	Chemical synthesis	SKWQHQQDSCRKQLQGVNLTPCEKHIMEKIQGRGDDDDDDDDD	MCF-7 and MDA-MB-231 cells were treated with 0–320 µM of peptides	Cell viability, migration, motility, invasion, activity and expression of MMP2 and MMP9↓	FAK/Akt/ERK and NF-κB signaling pathways↓	Jiang et al. (2016)
Lunasin	Chemical synthesis	SKWQHQQDSCRKQLQGVNLTPCEKHIMEKIQGRGDDDDDDDDD	Breast cancer cells were treated with 5, 10, 25, 50, 100 and 200 µM lunasin	IC_50_: 153 μM for MCF-7; 232 μM for MDA-MB-231 Inflammatory mediators: COX-2↓, IL-6↓ ERα↓, aromatase, VEGF↓, ERβ↑ Dead cells, low vitality cells↓ Health cells↑ Early apoptosis, late apoptosis↓ Cell viability (MCF-10A): not affected	Inflammation, angiogenesis, ER signaling↓	Hsieh et al. (2023)
Lunasin	Gene editing	SKWQHQQDSCRKQLQGVNLTPCEKHIMEKIQGRGDDDDDDDDD	MDA-MB-231 cells were treated with lunasin containing soybean powder	Cell proliferation↓		Hao et al. (2020)
Lunasin	Chemical synthesis	SKWQHQQDSCRKQLQGVNLTPCEKHIMEKIQGRGDDDDDDDDD	MDA-MB-231 cells were treated with 1, 10, and 25 µM lunasin	Cell proliferation↓ Early and late stages of apoptosis↑ ERBB2, AKT1, JUN and RAF1 mRNA↓	Causes an arrest in the S-phase	Hsieh et al. (2011)
Lunasin	Chemical synthesis	SKWQHQQDSCRKQLQGVNLTPCEKHIMEKIQGRGDDDDDDDDD	MDA-MB-231 cells were treated with 0.5, 1, 2.5, 5, 10, 25 and 50 µM lunasin	Histones H3 and H4 acetylation↓ Cell proliferation↓ IC_50_: 181 µM Cyclin D1, cyclin D3, CDK4 and CDK6↓	Triggers G1/S phase cell cycle arrest and induces apoptosis	Hernández-Ledesma et al. (2011)
Lunasin	Chemical synthesis	SKWQHQQDSCRKQLQGVNLTPCEKHIMEKIQGRGDDDDDDDDD	4T1 cells were treated with 1, 5, 10, 25 and 50 µM lunasin	Cell viability↓ Cell Metastasis, VEGF↓		Hsieh et al. (2016)
Lunasin	Chemical synthesis	SKWQHQQDSCRKQLQGVNLTPCEKHIMEKIQGRGDDDDDDDDD	MDA-MB-231 cells were mixed with 0.0625, 0.125, 0.25, 0.5, 1.0 mg/mL lunasin	Cell proliferation inhibition: 56.8% (1.0 mg/mL) CASP 3, 7, and 14 mRNA↑ Bax/Bcl-2↑ DNA replication: POLA1, POLA2, PRIM1, and PRIM2↓	Lysosome-mitochondrial axis↑	Hao et al. (2022)
Rapeseed	Rapeseed was fermented with *Bacillus subtilis* and *A. elegans*	WYP	MCF-7 cells were treated with 50, 100, 200, 400, 800, and 1600 μg/mL peptide mixtures	Cell viability↓ p53. bax↑, bcl-2↓	Mitochondria mediated apoptosis pathway↑	Wang et al. (2016)
Rapeseed	Rapeseed was fermented with *Bacillus subtilis* and *A. elegans*	Peptide extracts	MCF-7 cells were treated with 50, 100, 200, 400, 800, 1200, 1600, 2400, 3200 and 4800 μg/mL peptide mixtures	Cell proliferation↓		Xie et al. (2015)
Rapeseed	Alkalase 2.4 L and Thermoase PC10F were used to process rapeseed protein at a ratio of 3%–5% and pH of 8	<30 kDa	MCF-7 cells were treated with 3.75–20 mg/mL peptide and oligosaccharide extracts	Cells proliferation decreased by 80% at a sample concentration of 20 mg/mL IC_50_: 8.4 mg/mL		Ferrero et al. (2023)
Rapeseed	Alkalase 2.4 L and Thermoase PC10F	Composed of 2, 5 and 10 kDa peptides	MCF-7 cells were treated with 3.75–10 mg/mL peptide mixtures	Cells proliferation decreased by 83.9%		Ferrero et al. (2021)
Walnut	1.0% trypsin, 22h, 55°C	<10 kDa	MCF-7 cells were treated with 250, 500 and 1000 μg/mL peptide mixtures	Cell viability↓ Apoptosis, ROS↑ S phase arrested cells↓, G0/G1 phase arrested cells, depolarized cells↑; caspase-3, caspase-8, and caspase-9↑	Induced cell cycle arrest; mitochondria-mediated cell apoptosis pathway	Liao et al. (2016)
Walnut	Alkaline, papain, pepsin, trypsin	CTLEW	MCF-7 cells were treated with peptide fractions	Cell viability↓ IC_50_: 0.449–2.048 mg/mL Apoptosis increased from 4.07% to 18.11%↑ LC3-I↓, LC3-II↑	Immune activation	Ma et al. (2015)
Walnut	Chymotrypsin, trypsin, and proteinase K were used separately at 37°C and pH 8	<10 kDa, 5–10 kDa, 3–5 kDa and <3 kDa fractions	MDA-MB-231 cells were mixed with 100, 200, 300, 400, 500, 600, 700, 800, 900 and 1000 mg/mL peptide fractions	Cell viability↓		Jahanbani et al. (2016)
Olive	Thermolysin	LLPSY	MDA-MB-468 cells were treated with 50, 75, 150, 300 and 500 μg/mL of LLPSY	IC_50_: 97.6 ± 1.9 μg/mL for MDA-MB-468 cells Adhesion and migration↓ G2/M phase cells↑	Induced cell cycle arrest	Vásquez-Villanueva et al. (2018)
Chia	Pepsin and Pancreatin^®^	KLKKNL	MCF-7 cells were treated with 0.25, 0.5, 0.75, and 1 mg/mL of peptide fractions	Cell viability↓		Quintal-Bojórquez et al. (2021)
Rice bran	Chemical synthesis	EQRPR	MCF-7 and MDA-MB-231 cells were treated with 1 mg/mL of peptide	Cell viability↓ DNA fragmentation↑ Caspase-3, caspase-7, p53↑	p53 pathway↑	Li et al. (2014a)
Rice bran	Chemical synthesis	EQRPR	MCF-7 and MDA-MB-231 cells were treated with 50, 100, 200, 400, 500, and 1000 μg/mL of EQRPR	Cell viability↓ Caspase-8, Caspase-9↑ Bax↑, Bcl2↓ Fas↑, ErbB-2↓	Caspase-and mitochondrial-dependent pathway↑	Li et al. (2014b)
Corn gluten meal	1.8% alkaline protease, 16h, pH 5.5	Composition: Arg = 22.8 mg/g, Ala = 90.1 mg/g, Asp = 52.5 mg/g, Cys = 5.8 mg/g, Glu = 187.6 mg/g, Gly = 23.0 mg/g, Pro = 79.9 mg/g, Ser = 56.1 mg/g, Tyr = 41.3 mg/g	Sprague-Dawley rats	Mammary tumor incidence↓		Yamaguchi et al. (1997)
Zein	Alcalase^®^ CLEA	LALLALLRLRRRATTAFIIP	MDA-MB-231 cells	Migration decreased by around 50% Dead cells↑, Normalized metabolic activity↓		Trinidad Calderón (2022)
Buckwheat	Peptide was purified by DEAE-Sepharose FF anion exchange, Sephadex G-100 gel filtration and Sephacryl S-200 gel filtration column chromatography	Peptide extracts	Bcap37 cells were added with 10 μg/mL	Morphology changes occurred G0/G1 phase cells↑; G2/M phase cells↓ Bcl-2↓, Fas↑		Guo et al. (2010)
Barley	Ion Exchange Column Chromatography, Immunoaffinity Column Chromatography, Gel Electrophoresis	SKWQHQQDSCRKQLQGVNLTPCEKHIMEKIQGRGDDDDDDDDD	MCF-7 cells were added with 100 μM of lunasin	Histone acetylation↓		Jeong et al. (2002)
Spirulina platensis	Trypsin, Alcalase 2.4 L, papain	YGFVMPRSGLWFR	MCF-7 cells were added with 31.25–500 μg/mL of peptide fractions	Cell viability inhibition: 74%–88% IC_50_: 220.24 μg/mL for trypsin hydrolysate; 64.59 μg/mL for alcalase hydrolysate and 114.9–143.56 μg/mL for papain hydrolysate		Wang and Zhang (2016b)
Spirulina platensis	Alcalase; papain	AGGASLLLLR, LAGHVGVR, and KFLVLCLR	MCF-7 cells were added with 31.25–500 μg/mL of peptide fractions	IC_50_: 31.25 μg/mL Cell inhibition rate: 39.10% at 510 μg/mL for AGGASLLLLR; 36.09% at 620 μg/mL for LAGHVGVR Tumor volume↓		Wang and Zhang (2016a)
Spirulina platensis	Chemical synthesis	HVLSRAPR	MCF-7 cells were added with 500 μg/mL of peptide	Cell viability↓		Wang and Zhang (2017)
Cyanobacterium	Repeated normal- and reversed-phase silica gel column chromatography followed by HPLC	LIVFP	MCF-7 cells	Colony inhibition↓		Naman et al. (2017)
Cyanobacterium	LC-MS profiling and silica gel column chromatography, HPLC	PIAPPGFAF, APPGFAFPI, PPGFAFPIA	MCF-7 cells were treated with various concentrations of peptide mixtures	IC_50_: 0.58 μM		Lopez et al. (2016)
Porphyra haitanesis	Chemical synthesis	KKAAE	MCF-7 cells were treated with 0–500 ng/mL of peptide	Cell proliferation: 60% at 500 ng/mL p21, p27↑, cdk6, cyclin E↓; apoptosis↑ AKT, p-AKT, IGF-IR, ERK, p-ERK↓, P13K, IRS-I, SHC remained steady	PI3K-Akt pathway, IGF signaling pathway↓	Park et al. (2014)
Porphyra haitanesis	pH (6.5–8.5), temperature (35°C–55 °C), trypsin to substrate ratio (E/S; 1%–5%) and reaction time (2–10 h)	VPGTPKNLDSPR, MPAPSCALPRSVVPPR	MCF-7 cells were added with 100–500 μg/mL or 1 mg/mL of peptide fractions	IC_50_: 195.45–281.71 μg/mL apoptosis, G0/G1↑, G2/M↓	Induced G0/G1 cell cycle arrest	Fan et al. (2017)
Porphyra haitanesis	Pepsin, papain	QTDDNHSNVLWAGFSR	MCF-7 cells were added with 100–500 μg/mL of peptide fractions	IC_50_: 63.64 and 195.45 μg/mL		Mao et al. (2017)
Mistletoe Phoradendron tomentosum	Sephadex G-25 column, SP-Sephadex C25, HPLC	IISGTKCDSGWTH	Breast carcinoma	IC_50_: 87 nM-2.1 μM		Johansson et al. (2003)
Amaranth seed	Pepsin and pancreatin	ASP contained Ile and Leu ASP-D contained Thr, Ile, Lys and Leu ASP-HD contained Thr, Try, Phe, Lys and Leu	MDA-MB-231 cells were added with 20, 50, 100, 200 and 500 μg/mL of peptide fractions	IC_50_: 48.3 ± 0.2 μg/mL; apoptosis↑; caspase 3↑; migration↓		Taniya et al. (2020)
Amaranthus cruentus	Proteolytic enzyme (enzyme to substrate ratio (E/S) of 1:100), digested (4 h)	Peptide mixtures	MCF-7 cells were added with 7.8–1000 μg/mL of peptide fractions	Cell viability↓; apoptosis↑; Caspase-3/7↑		Ramkisson et al. (2020)
Dendrobium catenatum Lindley	Alcalase 2.4L, alcalase 37017 and trypsin	RHPFDGPLLPPGD, RCGVNAFLPKSYLVHFGWKLLFHFD and KPEEVGGAGDRWTC	MCF-7 cells were added with 50–500 μg/mL of peptide	Cell inhibition: 41.80% for RHPFDGPLLPPGD, 30.02% for RCGVNAFLPKSYLVHFGWKLLFHFD, and 39.31% for KPEEVGGAGDRWTC		Zheng et al. (2015)
*Matricaria chamomilla flowers*, *Cressa cretica aerial parts*, and *Ziziphora clinopodioides* leaves	Pancreatin enzyme (pH 7.5, 4h, 37°C)	Peptide mixtures	MCF-7 cells were added with 25, 50, 75, 100, 125, 150, and 175 μg/mL of peptide fractions	IC_50_: 82.42–138.6 μg/mL		Taghizadeh et al. (2020)
*Brucea javanica*	1% protein substrate concentration, 1:10 pepsin/substrate, 48h, 37°C	≤3 kDa	MCF-7 cells were added with 0.03125, 0.0625, and 0.125 μg/mL of peptide fractions	IC_50_: 0.25–33.68 μg/mL; apoptosis↑ G0/G1↑, G2/M↓ P53, PTEN, and NM23-H1↑	p53 pathway↑	Shi et al. (2022)
Black seeds (Nigella sativa)	FPLC, Hiload™ 16/60 Superdex™ 200 pg gel filtration column	5–150 kDa, 15–100 kDa, 5–15 kDa	MCF-7 cells were added with 25, 50, 100, 200, and 400 μg/mL of peptide fractions	IC_50_: 8.05–16.00 μg/mL; BAX, Caspase-3 and BCL-2↑; SURVIVIN↓		Khurshid et al. (2020)
Zingiberaceae plant rhizomes	Pepsin and pancreatin	Peptide mixtures	BT474 cells were added with 0–25 μg/mL of peptide fractions	Not affected		Inthuwanarud et al. (2016)

MMP, matrix metalloproteinases; FAK, focal adhesion kinase; ERK, extracellular signal-regulated kinase; NF-κB, nuclear factor-κB; COX-2, cyclooxygenase-2; IL-6, Interleukin-6; ER, estrogen receptor; EGF, vascular endothelial growth factor; CASP, caspase; SDS-PAGE, sodium dodecyl sulfate-polyacrylamide gel electrophoresis; FPLC, fast performance liquid chromatography; IGF, Insulin-like growth factor; AKT1, protein kinase B; PI3K-Akt, phosphoinositide 3-kinase/Akt; GOT, glutamic-oxaloacetic transaminase; GPT, glutamic-pyruvic transaminase; ERBB2, human epidermal growth factor receptor 2; RAF1, rubisco accumulation factor 1; POLA1, polymerase alpha 1; POLA2, polymerase alpha 2; PRIM1, primase catalytic domain 1; PRIM2, primase catalytic domain 2; ROS, reactive oxygen species; PTEN, phosphatase and tensin homolog deleted on chromosome 10.

##### 3.1.2.1 Anti-breast cancer effects of lunasin

One of the most well-known anticancer peptides from legume proteins is lunasin , which was initially discovered in soybeans and has recently been identified in wheat, barley, and other seed proteins (Jeong et al., 2010). Research by some scholars has found that lunasin can effectively reduce the viability, motility, and invasiveness of MCF-7 and MDA-MB-231 cells, and decrease the expression of MMP2 and MMP9, which was attributed to the downregulation of the FAK/Akt/ERK and NF-κB signaling pathways (Jiang et al., 2016). The carboxyl-terminal of lunasin contains 8 Asp residues, which have been found to exhibit antimitotic activity when bound to hypoacetylated chromatin regions, such as those found in centromeres. Thus, the kinetochore complex forms improperly, and the microtubules cannot attach to the centromeres, causing mitotic arrest and ultimately, the death of cancer cells (Hernández-Ledesma et al., 2009). Recently, there has also been research on the synergistic effects of lunasin with other active substances. For instance, Hsieh et al. (2011) found that lunasin can synergize with anacardic acid to produce a stronger anti-breast cancer effect, manifesting in the fact that the combination of them induced stronger toxicity to breast cancer cells, inhibited the growth of breast cancer cells more efficiently, led to more cell cycle arrest and apoptosis, and more significantly regulated the expression of genes related to cell cycle and DNA damage repair, such as CCNE1, CDK2, CDK4, and E2F1.

##### 3.1.2.2 Anti-breast cancer effects of other legume protein-derived peptides

In addition to lunasin, other anticancer peptides consisting of 2–20 amino acids have also been identified from chickpeas, lentils, etc. For example, Lam and Ng (2011) and Xue et al. (2015) identified two anti-breast cancer peptides from chickpeas, namely, ANDISFNFVRFNETNLILGG and RQSHFANAQP. They both found that these peptides significantly promoted apoptosis and inhibited the proliferation of breast cancer cells. Lam and Ng (2011) discovered that ANDISFNFVRFNETNLILGG produced anticancer effects by causing breast cancer cells to remain in the G2/M cycle and activating the death receptor-mediated pathway. Xue et al. (2015) found that the anti-breast cancer mechanism of RQSHFANAQP is reflected in causing a decrease in reactive oxygen species (ROS) levels and regulation of the tumor protein p53 signaling pathway. Additionally, lentils are also an important source of anticancer peptides among legume proteins. Kuerban et al. (2020) obtained lentil protease hydrolysis peptide mixtures with different molecular weights, among which the < 3 kDa component had the highest anti-breast cancer proliferation effect, with an IC_50_ value of 12.27 mg/mL and 27 active peptides were identified from the mixtures. However, this study did not synthesize part of the active peptides and further study their anti-breast cancer capabilities, so we cannot conclude that all 27 active peptides are anti-breast cancer peptides. The anti-breast cancer effects of many legume protein-derived peptides, such as those from fava beans and red beans, have not received sufficient attention, and more efforts can be invested in this area in the future.

### 3.2 Oil crop protein-derived anti-breast cancer peptides

#### 3.2.1 Characteristics of oil crop proteins

Due to the ability to provide oils and proteins at a low cost, the importance of oil crops has been widely recognized globally (Hossain et al., 2019). Generally, the protein content of oil crops is lower than that of legumes but higher than that of grains (Table 1). However, the protein content of defatted oilseed crops significantly increases after oil extraction. For example, the protein content of rapeseed is 17%–26%, but it increases to 51%–54% after the oil is removed (Östbring et al., 2019). Therefore, oil crops are an ideal by-products in the food industry after oil extraction and they become important sources of bioactive peptides after enzymatic hydrolysis or fermentation.

Other oil crops, such as walnuts (protein content: 18%–24%), olives (protein content: 1.6%), sunflower seeds (protein content: 20.78%), sesame seeds (protein content: 25%), coconut flour (protein content: 33%), and chia seeds (protein content: 26%), can also be used to extract bioactive peptides. For example, antioxidant peptides and ACE-inhibiting peptides are extracted from defatted walnuts (Gu et al., 2015; Chen et al., 2020), lipid-lowering peptides from defatted olive seeds (Prados et al., 2020), low-sodium peptides from sunflower meal (Guo et al., 2019), and anti-diabetic and anti-obesity active peptides from black sesame meal (Chaipoot et al., 2023).

#### 3.2.2 Anti-breast cancer activities of oil crop protein-derived peptides

In recent years, the anti-breast cancer effects of bioactive peptides derived from oil crops have received widespread attention. Many bioactive peptides, such as WYP (rapeseed protein) (Wang et al., 2016), LLPSY (olive seed protein) (Vásquez-Villanueva et al., 2018), and KLKKNL (chia seed protein) (Quintal-Bojórquez et al., 2021), have been identified from fermented or enzymatically hydrolyzed oil crop proteins and have demonstrated anticancer activity in cellular or animal breast cancer models. In addition to individual active peptides, researchers have also studied the anti-breast cancer effects of mixed peptides from oil proteins, including mixtures from walnut, olive, and chia seed proteins (Jahanbani et al., 2016; Vásquez-Villanueva et al., 2018; Quintal-Bojórquez et al., 2021). The anti-breast cancer peptides and mixed peptides identified from the aforementioned oil crops have been demonstrated to effectively reduce breast cancer cell proliferation, adhesion, and migration, promote ROS accumulation, induce breast cancer cell apoptosis, and cause overexpression of apoptosis-related proteins (Liao et al., 2016). Moreover, studies have shown that these peptides exert their effects by activating the mitochondrial apoptosis pathway and inducing cell cycle arrest (Wang et al., 2016; Vásquez-Villanueva et al., 2018).

Wang et al. (2016) used *Bacillus subtilis* and *Actinomucor elegans* to ferment rapeseed protein and applied the resulting rapeseed peptides to a human breast cancer cell model. The research showed that rapeseed peptides effectively reduced the viability of MCF-7 cells from 100% to 69.38%. In the rapeseed peptide mixture, an anti-cancer peptide (WYP) was identified, which played a role in regulating the expression of proteins of the mitochondrial apoptosis pathway, characterized by the downregulation of Bcl-2 protein levels and the upregulation of p53 and Bax protein expressions. Another study reported by Ma et al. (2015) hydrolyzed walnut protein with alkaline, papain, pepsin, and trypsin, ultimately obtaining a peptide with high anti-breast cancer activity, CTLEW, from the peptide fractions. The results showed that CTLEW maintained up to 82.1% of its anti-breast cancer activity after digestion by gastric protease, trypsin, and bile, and could significantly induce cell apoptosis (cell apoptosis rates increased from 4.07% to 18.11%) and autophagy (LC3-I expression decreased, LC3-II expression increased), as well as disrupt the cell cycle (sub-G1 phase cells increased from 7.67% to 18.24%). This may be associated with CTLEW’s effects on the activation of spleen lymphocytes and macrophages. Overall, this study is a very well-established study involving anticancer peptide screening, identification and activity measurement. Similarly, Vásquez-Villanueva et al. (2018) found that the proteolytic products of olive seed protein were rich in antihypertensive and anticancer peptides, such as VVLED, VSVDD, LGLGD, LSEAEK, ALMSPH, LMAPH, LLPSY. Among them, LLPSY demonstrated a strong anti-proliferative effect on breast cancer cells, with an IC_50_ value of 97.6 ± 1.9 μg/mL, and also exhibited effects against breast cancer cell adhesion and migration, causing breast cancer cell arrest in the G2/M phase. However, this study did not explore the action mechanism of LLPSY and its anti-breast cancer effects in *in vivo* models, resulting in the impossibility of applying LLPSY as a functional food ingredient or clinical drug temporarily. Currently, research on identifying anticancer peptides from oil crop proteins and verifying their anticancer activity is not very extensive. More efforts are needed in mechanism studies and animal models in the future.

### 3.3 Cereal protein-derived anti-breast cancer peptides

#### 3.3.1 Characteristics of cereal proteins

Cereals are kinds of edible seeds or grains of grass family plants, which constitute a large portion of the food pyramid and serve as a major source of energy in the human diet (Sudheesh et al., 2022). In developed countries, diets typically include a wide range of dietary protein origins, and thus the compositional and nutritional deficiencies of individual dietary components do not impact the overall intake of nutrients. However, some less-developed countries tend to rely predominantly on cereals (Shewry, 2007). Although cereals are a primary source of carbohydrates, proteins, vitamins (such as B vitamins), and minerals, their protein content is lower than that of oilseeds and legumes, and they lack certain essential amino acids (such as lysine, threonine, and tryptophan), which may lead to nutritional imbalances (Gulati et al., 2020; Sudheesh et al., 2022). Scientists have made several good recommendations for addressing the amino acid deficiencies in cereal proteins, such as combining cereal proteins with legume proteins that are rich in lysine and low in sulfur-containing amino acids, thereby significantly improving protein utilization (Temba et al., 2016; Sudheesh et al., 2022).

Common cereals include rice (protein content 6.3%–15%), corn (protein content 6%–12%), wheat (protein content 12%), barley (protein content 8%–30%), millet (protein content 7%–12%), and buckwheat (protein content 6%–12%), etc (Table 1). Storage proteins occupy a major proportion of cereal proteins. Based on their solubility in water, salt solutions, dilute alcohol, and dilute acid/alkali, grain storage proteins can be categorized into four types: albumins, globulins, prolamins, and glutelins (Sudheesh et al., 2022). Most cereal proteins exhibit poor processing characteristics, making them difficult to use directly in food products, and therefore, they usually require further reprocessing (Gong et al., 2022). Bioactive peptides provide a direction for in-depth processing of cereal proteins. After digestion, enzymatic hydrolysis, or fermentation, cereal proteins can produce a large number of bioactive peptides, which exhibit excellent antioxidant, antihypertensive, antidiabetic, anticancer, and anti-inflammatory properties in the body (Gong et al., 2022).

#### 3.3.2 Anti-breast cancer activities of cereal proteins-derived peptides

Anticancer peptides in cereal proteins have been extensively studied, such as Glu-Gln-Arg-Pro-Arg isolated from rice bran protein showing activity against liver and colon cancer (Kannan et al., 2010; Gasymov et al., 2021), anticancer peptides of LRQQ, QLQGV, WQPN, GLQDL, AMCGVV, QGVAAA, LRQQ, YLRQ, AQVAQ, QLQGV, TPCATS, QQLQ, WQPN from sorghum kafirin (Xu et al., 2019), and colon cancer peptides of FHPFPR, NWFPLPR, and HYNPYFPG isolated from quinoa protein (Fan X. et al., 2022). However, peptides against breast cancer have not been widely explored in cereal proteins, with only a few such as rice bran (Li et al., 2014b), corn gluten meal (Yamaguchi et al., 1997), zein (Trinidad Calderón, 2022), buckwheat (Guo et al., 2010), and barley (Jeong et al., 2002) peptides being identified for their anti-breast cancer activities. These breast cancer peptides are mainly obtained through chemical synthesis, alkaline protease treatment, and purification using tools like diethylaminoethyl-Sepharose fast flow anion exchange and Sephadex G-100 gel filtration to obtain either anticancer peptide monomers or mixtures, which exhibit anticancer effects in *in vitro* breast cancer cell models or rat breast cancer models.

For instance, Li et al. (2014a) and Li et al. (2014b) isolated an anticancer peptide, EQRPR, from rice bran protein and found that it exhibited promising anticancer effects in MCF-7 and MDA-MB-231 breast cancer cell models, characterized by a significant reduction in breast cancer cell viability and a dramatic upregulation of DNA fragmentation. Additionally, the expression of pro-apoptotic proteins such as caspase-3, caspase-7, caspase-8, caspase-9, and bax was significantly increased, while the expression of Bcl-2 decreased, which may be related to the activation of the mitochondria-dependent apoptosis pathway and the p53 tumor suppressor pathway. However, these studies paid less attention to indicators of breast cancer cell inhibition, such as active peptides’ effects on breast cancer cell migration, adhesion, and invasion, and did not use animal breast cancer models to verify the effects and mechanisms of anticancer peptides.

As reported in the research by Yamaguchi et al. (1997), peptides hydrolyzed from corn gluten meal were found to effectively reduce the incidence of mammary tumors in Sprague-Dawley rats. However, this paper did not identify the specific active peptides in the corn gluten meal hydrolysate nor did it explore the action mechanisms of peptides or peptide fractions against breast cancer. LALLALLRLRRRATTAFIIP obtained from the Alcalase^®^ CLEA enzymatic hydrolysis product of zein showed excellent inhibition effects on breast cancer cells and reduced breast cancer migration rate to 50% (Trinidad Calderón, 2022). Additionally, a significant decrease in normalized metabolic activity of MDA-MB-231 cells was observed after zein peptide treatment. However, the paper did not explore the mechanism of action of LALLALLRLRRRATTAFIIP, making its application in clinical anti-breast cancer drugs still a long way off.

In the Bcap37 breast cancer cell model, the buckwheat protein bioactive peptide extracts significantly altered the morphology of breast cancer cells, causing severe cell cycle arrest in the G0/G1 phase, and significantly downregulating Bcl-2 protein content while upregulating Fas protein content, thus exerting anti-breast cancer effects (Guo et al., 2010). Some scholars have also identified lunasin from barley seeds, which has been shown to significantly reduce histone acetylation processes in breast cancer cells (Jeong et al., 2002). However, both studies were very superficial, not involving mechanism exploration or animal model verification, indicating that currently, the number and types of active peptides identified from cereal proteins are limited, and the active effects and mechanisms of these peptides are unclear, leading to the neglect of the importance of anti-breast cancer active peptides isolated from cereal protein. In the future, we can promote the development of cereal protein-derived active peptides not only from the perspective of strengthening the action mechanism of anti-breast cancer peptide mixtures but also from the perspective of purifying the hydrolysis products of cereal proteins in order to obtain high anti-breast cancer active peptides.

### 3.4 Marine plant protein-derived anti-breast cancer peptides

#### 3.4.1 Characteristics of marine plant proteins

Marine biological resources are an important source of bioactive compounds with both industrial and nutritional potential (Kang et al., 2015). Approximately 70% of the Earth’s surface is coated by oceans, which comprise 90% of the biosphere (Cheung et al., 2015). Marine species account for about half of global biodiversity, with an estimated 2210000 species, of which only around 190000 have been documented (Kang et al., 2019). Due to the challenges of exploring deep-water habitats, many marine biological compounds remain isolated, unidentified, and uncharacterized (Cheung et al., 2015). Thus, there is an urgent need for further development of living marine resources.

Marine plant communities, involving microalgae, macroalgae (seaweeds), and flowering plants (mangroves and other halophytes), account for over 90% of marine biomass (Boopathy and Kathiresan, 2010). Historically, marine plants have been widely used for medicinal purposes in India, China, and Europe, such as using seaweeds to treat infectious diseases and inflammation (Hwang et al., 2022), and using seaweed to address obesity, diabetes, and hypertension (Ikeda et al., 2003; Li et al., 2020; Li et al., 2021). Marine plant protein hydrolysate is rich in bioactive peptides and free amino acids and serve as an important resources for cancer prevention (Ahmed et al., 2021). Among them, bioactive peptides were first discovered and isolated in marine species, and their biological activity depends on their amino acid composition and sequence, including neuromodulatory peptides, antioxidant peptides, antiviral peptides, antitumor peptides, and antimicrobial peptides (Ahmed et al., 2021). With their extensive biological activity, low allergenicity, and low toxicity, marine peptides have shown high potential nutritional and medicinal value, attracting the interest of the pharmaceutical industry (Cheung et al., 2015). Some pharmaceutical companies have successfully extracted and purified numerous active peptides from marine plants for the treatment or prevention of various diseases. For instance, Dermochlorella DG, which main active components are oligopeptides purified from algae, has entered the cosmetic market in the form of skin-firming and toning products (Cheung et al., 2015). Two peptides isolated from spirulina hydrolysate, namely, P1 (LDAVNR) and P2 (MMLDF), have exhibited protective effects against early arteriosclerosis in endothelial cells (Cheung et al., 2015). Additionally, compounds like Bellamine A (a tetrapeptide), Symplostatin, Dolastatin 10 and 15 (pentapeptides) are isolated from cyanobacteria, exhibiting significant *in vivo* anticancer effects (Ahmed et al., 2021). Marine anticancer peptides generally participate in several cellular and molecular pathways, such as DNA defense, regulation of cell cycle, activation of apoptosis, inhibition of angiogenesis, and tumor migration, invasion, and metastasis, thereby exerting their anticancer efficacy (Ahmed et al., 2021).

#### 3.4.2 Anti-breast cancer activities of marine plant protein-derived peptides

The anti-breast cancer activity of marine plant protein-derived bioactive peptides is increasingly attracting attention. Spirulina protein, one of the most notable marine plant proteins, contains a high protein content of 60%–70% (Saranraj and Sivasakthi, 2014). Various anti-breast cancer peptides such as YGFVMPRSGLWFR, AGGASLLLLR, LAGHVGVR, KFLVLCLR, and HVLSRAPR have already been identified from spirulina protein (Wang and Zhang, 2016a; b; 2017). Wang and Zhang (2016b) identified a spirulina protein peptide, YGFVMPRSGLWFR, and applied it to the MCF-7 breast cancer cell model. Results recorded that the active peptide could inhibit the growth of breast cancer cells by 46.68% at a concentration of 500 μg/mL. However, this study focused on the broad anticancer activity of spirulina protein peptides, rather than specifically on anti-breast cancer activity, leading to a neglect of exploring the action mechanisms against breast cancer. Wang and Zhang (2016a) also identified another three anti-breast cancer peptides from spirulina protein, AGGASLLLLR, LAGHVGVR, and KFLVLCLR, with AGGASLLLLR inhibiting breast cancer cells by 39.10% at a concentration of 510 μg/mL and LAGHVGVR by 36.09% at 620 μg/mL. Additionally, a mixture of spirulina protein peptides showed stronger inhibition effects on breast cancer, with an IC_50_ value of 31.25%, surpassing that of individually isolated pure active peptides from spirulina, which was likely due to a synergistic effect between the peptides and amino acids (Zhang et al., 2017; Liu et al., 2018; Du et al., 2019). Similarly, the anti-breast cancer activity of Cyanobacterium has also garnered attention, with Naman et al. (2017) and Lopez et al. (2016) successfully identifying active peptides such as LIVFP, PIAPPGFAF, and APPGFAFPI from it, which not only inhibited the growth of MCF-7 cells but also disrupted the colony of breast cancer cells. *Porphyra haitanesis*, another important marine plant protein source, has a protein content of 28%–39% (Grossmann et al., 2020), yielded peptides such as KKAAE (Park et al., 2014), VPGTPKNLDSPR, MPAPSCALPRSVVPPR (Fan et al., 2017), and QTDDNHSNVLWAGFSR (Mao et al., 2017), which suppressed proliferation and promoted apoptosis of breast cancer cells, with KKAAE showing a prominent anti-proliferative effect (inhibition rate: 60%at a concentration of 500 ng/mL) compared to other *Porphyra hairiness-*derived peptides. The anti-breast cancer proliferative effect of *P. haitanesis* peptides might be related to cell cycle arrest and the activation of the PI3K-Akt and IGF signaling pathways. These studies not only isolated pure peptides but also explored their anti-breast cancer mechanisms, providing insights into the isolation of peptides from marine protein and the validation of their anti-breast cancer effects and mechanisms. However, consistent with the low utilization of marine resources, current exploration of marine plant protein-derived bioactive peptides is still scarce, with conventional research focusing on peptides from spirulina, Cyanobacterium, and *P. haitanesis*, while the anti-breast cancer activity of peptides from eelgrass, mangroves, and marine lettuce remains unexplored. Additionally, beyond active peptides, some glycopeptides and cyclic peptides may exhibit more significant anti-breast cancer activity. However, current research not only rarely focuses on these structurally unique peptides, but the relationship between their structure and anti-breast cancer activity has also not been elucidated.

### 3.5 Other plant protein-derived anti-breast cancer peptides

In addition to the above anti-breast cancer peptides, there are also some other plant protein sources of anti-breast cancer peptides that have been brought to the attention of scholars. Overall, anti-breast cancer peptides from vegetable and fruit protein sources have received relatively less focus. In one of the few studies, Taniya et al. (2020) and Ramkisson et al. (2020) found that amaranth seed protein hydrolysates could effectively inhibit the activity of breast cancer cells and promote the expression of pro-apoptotic proteins (caspase-3 and caspase-7). However, these studies did not identify the active peptides in amaranth seed protein hydrolysates. Moreover, flowers and leaves, although uncommon, can also be used to extract anti-breast cancer peptides. Zheng et al. (2015) obtained several anti-breast cancer peptides from *Dendrobium catenatum* Lindley, which are RHPFDGPLLPPGD, RCGVNAFLPKSYLVHFGWKLLFHFD, and KPEEVGGAGDRWTC; these peptides exhibited 41.80%, 30.02%, and 39.31% inhibitory activity against MCF-7 cells, respectively. The study of Taghizadeh et al. (2020) utilized three plants, namely, *Matricaria chamomilla* flowers, *Cressa cretica* aerial parts, and *Ziziphora clinopodioides* leaves, all showing high anti-breast cancer activity with IC_50_ values ranging from 82.42 to 138.6 μg/mL. However, these studies did not explore the anti-breast cancer mechanisms of plant-derived peptides. *Brucea javanica* protein hydrolysates with peptide fractions smaller than 3 kDa also exhibited anti-proliferative and apoptotic activities in breast cancer cells, blocked the cell cycle arrest, and promoted the expression of proteins such as p53, phosphatase and tensin homologue (PTEN), NM23-H1, which all related to the activation of the p53 signaling pathway (Shi et al., 2022). Overall, there are relatively few studies using nude or mouse breast cancer cell models to explore the anti-breast cancer activity of PDBP. Additionally, many more food-derived plant proteins have yet to be studied for screening anti-breast cancer peptides, such as almond protein, potato protein, wheat bran, and spinach protein.

## 4 Production, stability and applications of plant protein-derived anti-breast cancer peptides

### 4.1 Production of plant protein-derived anti-breast cancer peptides

The common methods for obtaining bioactive peptides from plant proteins are enzymatic hydrolysis and microbial fermentation (Daliri et al., 2017). Upon determining the structure of a bioactive peptide, the synthesis of the peptide can be achieved.

#### 4.1.1 Enzymatic hydrolysis

Enzymatic hydrolysis involves the incorporation of commercial enzymes or naturally isolated enzymes from biological sources into plant proteins to obtain bioactive peptides since these enzymes are responsible for cleaving the peptide bonds established within the proteins, thereby releasing the encapsulated peptides (Cruz-Casas et al., 2021). The advantages of enzymatic hydrolysis for producing anti-breast cancer peptides from plant proteins include short reaction times, minimal by-products, high product quality, energy savings, and ease of obtaining stable hydrolysis results, while the disadvantages include relatively high costs, susceptibility to enzyme inactivation, potential damage to some amino acids and the need for precise management of hydrolysis conditions (Cruz-Casas et al., 2021). Commonly used enzymes for producing anti-breast cancer peptides from plant proteins include alcalase, pepsin, trypsin, papain, thermolysin, chymotrypsin, and proteinase K (Table 2). Among these, alcalase, pepsin, and trypsin are the most prominent enzymes, which have been shown to release numerous anti-breast cancer peptides from sources such as soybean, moth bean seed, lentil, rapeseed, walnut, chia, corn gluten meal, zein, and *Spirulina platensis*, including peptides such as KTCENLADTY, CTLEW, KLKKNL, LALLALLRLRRRATTAFIIP, YGFVMPRSGLWFR, AGGASLLLLR, LAGHVGVR, and KFLVLCLR (Table 2). Wang and Zhang (2016a) employed 5% (w/w) alkaline protease (pH 8.5, 55°C, 5 h) followed by 4% (w/w) papain (55°C, pH 6.5, 3 h) to sequentially hydrolyze 3% of *Spirulina platensis* protein solutions, thereby successfully obtaining bioactive peptide fractions of <3, 3–5, 5–10, and >10 kDa. Among these, the <3 kDa peptide fraction exhibited the highest anti-breast cancer activity with an inhibition rate of 95%. In another study, they utilized three proteases: trypsin, with an enzyme-to-substrate ratio (E/S) of 3% (w/w), at 42°C, pH 8, for 8 h; Alcalase, with an E/S ratio of 6% (w/w), at 50°C, pH 8.5, for 8 h; and papain, with an E/S ratio of 4% (w/w), at 55°C, pH 6.5, for 8 h, to treat 3% protein solution of *Spirulina platensis*, and obtained peptide fractions with molecular weight of 3, 3–5, 5–10, and >10 kDa (Wang and Zhang, 2016b). Similarly, the <3 kDa peptide fraction nearly inhibited 100% breast cancer cells. These studies indicate that different hydrolysis conditions can yield bioactive peptide fractions with varying anti-breast cancer effects, and the use of more commercial proteases tended to produce active peptide mixtures with higher anti-breast cancer effects.

Thermolysin, papain and chymotrypsin are examples of other proteolytic enzymes that have been applied to release various anti-breast cancer peptides, such as LLPSY, YGFVMPRSGLWFR, AGGASLLLLR, LAGHVGVR, KFLVLCLR, QTDDNHSNVLWAGFSR, and CTLEW (Table 2). For instance, Vásquez-Villanueva et al. (2018) utilized Thermolysin (0.5 g enzyme/g protein ratio, pH 7.5, 50°C, for 2 h) to hydrolyze olive seed protein and subsequently collected the <3 kDa fraction, from which a highly anti-breast cancer active peptide, LLPSY, was identified. Finally, some researchers suggest that more than a single proteolytic enzyme (whether purified or crude) can be employed to hydrolyze plant proteins to produce protein hydrolysates containing short peptide sequences. We have found similar viewpoints in our research. For example, the bioactive peptides obtained from the trypsin-treated lentil hydrolysis include EVASYSGW, FFADTGIK, TSFFDPAGG, LPAKSSAPK, LHVGDTEK, ESTYGILD, VYKIEDM, LECTSCDK, DDHDLKR, RNGIIK, LAVNPKSLG, YILNYVN, PNIHYVR, VHVSLK, HNEQLEK, QVIDGIPN, QLQVLAGGL, EKLAVNPK, PLIRSLAK, KSISTFSK, TQTFGNET, NHGMHFR, and NQLLVNR, most of which consist of more than six amino acids. Similarly, Trinidad Trinidad Calderón (2022) exclusively used Alcalase^®^ CLEA to process zein and identified a bioactive peptide containing 20 amino acid residues (LALLALLRLRRRATTAFIIP). In contrast, the anti-breast cancer peptide of CTLEW identified from walnut after treatment with alkaline protease, papain, pepsin, and trypsin contained only five amino acids (Ma et al., 2015). Consequently, employing multiple commercial enzymes in conjunction for the processing of plant proteins contributes to obtaining bioactive peptides with excellent anti-breast cancer efficacy.

#### 4.1.2 Microbial fermentation

Microbial fermentation involves cultivating bacteria or yeast on protein substrates to enzymatically hydrolyze proteins using their enzymes during their growth phase. The proliferating microorganisms secrete their proteolytic enzymes into the plant protein to release anti-breast cancer bioactive peptides from the parent proteins (Daliri et al., 2017). Submerged fermentation and solid-state fermentation are the most widely used microbial fermentation processes methods currently. The former involves cultivating microorganisms in nutrient-rich liquid media, which facilitates the easier separation of bioactive peptides. The latter cultivates microorganisms in nutrient-rich solid substrates, which are well-suited for fungi and microorganisms that thrive in dry conditions (Cruz-Casas et al., 2021). The advantages of microbial fermentation technology include low cost, short production cycles, health and safety benefits, the ability to obtain fermented products with high bioactivity, and enhancements to the sensory, nutritional, and textural properties of these products (Cruz-Casas et al., 2021). However, it also has significant drawbacks, such as poor batch stability and susceptibility to contamination. Generally, appropriate substrates, suitable microorganisms, and optimal environmental conditions, such as pH, temperature, and humidity, contribute to the production of high-bioactivity peptides (Zhang Y. Z. et al., 2024). Among the widely used fermentative microorganisms, lactic acid bacteria stand out due to their high adaptability to various environments and plant and animal substrates, making them one of the most valuable microorganisms for obtaining anti-breast cancer bioactive peptides (Table 2). Chang et al. (2002) utilized various microorganisms, including *Lactobacillus acidophilus*, *Lactobacillus bulgaricus*, *Streptococcus lactis*, *Bifidobacteria*, and yeasts, to ferment soybean protein and isolated peptide fractions with molecular weight of <3, 3–10, and 10–50 kDa, with 10–50 kDa peptide fractions exhibiting the highest activity in inhibiting breast cancer cell proliferation. In addition to lactic acid bacteria, *Rhizopus sp. strain*, *Bacillus subtilis*, and *A. elegans* have also been employed to produce anti-breast cancer bioactive peptides. Factors such as the plant protein types, microorganism strains, fermentation pH, temperature, humidity, and fermentation time, all influence the anti-breast cancer activity of the plant protein peptide mixtures. For instance, Yeap et al. (2013) identified *Rhizopus sp.* strain 5351 as having the best fermentation capability for mung beans among various strains. Besides, optimal conditions for fermenting rapeseed with *Bacillus subtilis* and *A. elegans* were identified as pH 6.5, temperature of 35°C, and fermentation time of 2 days (Xie et al., 2015). Under these optimal fermentation conditions, the resultant plant peptide mixtures demonstrated the highest anti-breast cancer activity. However, research on obtaining anti-breast cancer bioactive peptide mixtures or peptide monomers from plant proteins using microbial fermentation is currently limited.

### 4.2 Stability of plant protein-derived anti-breast cancer peptides

Numerous PDBPs have already been identified from plant proteins via enzymatic hydrolysis methods, fermentation, and chemical and physical hydrolysis, and these peptides show certain stability against heat and acidic or alkaline conditions. However, their integrity might be destroyed when exposed to the gastrointestinal environment. Fortunately, research has found that PDBPs maintain their biological activity, even when fully hydrolyzed into smaller peptide fragments.

Generally, high temperatures can induce changes in the secondary structure of bioactive peptides, leading to aggregation and loss of stability (Pei et al., 2022). Some bioactive peptides, such as VLSTSFCPK, VLSTSFHPK, VLSTSFYPK, KAAAAP, AAPLAP, KPVAAP, IAGRP, and KAAAATP, can withstand temperatures exceeding 65°C, indicating their potential for use as functional food components (Singh and Vij, 2018). Interestingly, there are also viewpoints suggesting that heating can enhance the bioactivity of peptides, such as their antioxidant capacity and ACE activity (Singh and Vij, 2018). However, no studies have yet explored the relationship between thermal treatment and the anti-breast cancer activity of plant protein-derived bioactive peptides. According to Mirzaei et al. (2020), a higher percentage of β-sheet structures in peptides correlates with lower thermal stability. Moreover, bioactive peptides containing Cys and/or His generally exhibit relatively low thermal stability, primarily due to the high sensitivity of Cys to heat-induced aggregation via disulfide bonds and the susceptibility of His side chains to oxidation caused by heat treatment. Consequently, anti-breast cancer peptides such as chickpea protein peptide ANDISFNFVRFNETNLILGG, lentil protein peptides EVASYSGW, FFADTGIK, TSFFDPAGG, LPAKSSAPK, LHVGDTEK, rapeseed protein peptide WYP, olive protein peptide LLPSY, and chia protein peptide KLKKNL tend to exhibit higher thermal stability (Table 2). Nonetheless, there is currently limited research on the thermal stability and secondary structure of anti-breast cancer peptides derived from plant proteins, making it difficult to accurately assess their heat sensitivity. Addressing these challenges will facilitate the production of functional foods enriched with anti-breast cancer bioactive peptides.

pH conditions can alter the ionization characteristics of peptides and facilitate electron transfer, thereby affecting the quantity, size, structure, amino acid composition, and hydrophobicity of peptides, ultimately leading to changes in the stability of bioactive peptides (Singh and Vij, 2018; Mirzaei et al., 2020). Threonine, serine, and cysteine are unstable under alkaline conditions, while glutamic acid is unstable under acidic conditions (LARSEN, 1980). According to Table 2, most anti-breast cancer bioactive peptides may be acid-stable, such as RQSHFANAQP, TSFFDPAGG, and LPAKSSAPK, while a few may exhibit alkaline instability, such as CTLEW from walnut protein, KTCENLADTY from ground beans, and LLPSY from olive.

Oral administration is the most convenient and acceptable method for delivering plant protein-derived anti-breast cancer bioactive peptides, as it can enhance patient compliance and reduce discomfort (Pei et al., 2022). However, many of these peptides are digested by gastrointestinal enzymes such as trypsin, pepsin, chymotrypsin, and pancreatic enzymes into inactive peptide fragments or free amino acids, thus losing their therapeutic value in the body (Segura-Campos et al., 2011). Therefore, the resistance of plant protein-derived anti-breast cancer bioactive peptides to gastrointestinal proteolytic digestion is crucial for their bioactivity. Generally, anti-breast cancer bioactive peptides with lower molecular weights exhibit greater stability in the gastrointestinal tract and are more readily absorbed by the body, thus maximizing their efficacy (Zhang et al., 2017; Pei et al., 2022). For instance, lunasin is believed to degrade into small peptide fragments during gastrointestinal digestion, including SKWQHQQDSC, RKQLQGVN, VNLTPCEKHIME, LTPCEKHIME, KIQGRGDDDDDDD, and KIQGRGDDDDDDDDDDD (Indiano-Romacho et al., 2019). Consequently, examining the anti-breast cancer activity of these small peptide fragments may be more valuable than studying lunasin itself, although such research has yet to be conducted. From the perspective of the properties and amino acid composition of bioactive peptides, those containing higher proportions of acidic amino acids or exhibiting high hydrophobicity generally demonstrate greater stability against gastrointestinal digestion. Notably, research by Sontakke et al. (2016), Vermeirssen et al. (2004) and López-Barrios et al. (2014) has also demonstrated that peptides containing proline typically exhibit high resistance to degradation by digestive enzymes, thus maintaining excellent anti-breast cancer activity after oral absorption. Therefore, anti-breast cancer peptides such as WYP, LLPSY, and EQRPR may possess higher resistance to digestive enzyme degradation compared to other plant protein-derived anti-breast cancer peptides, making them more suitable for oral administration.

To ensure that plant protein-derived anti-breast cancer bioactive peptides retain their efficacy upon entering the human body, the following methods can be employed to enhance their stability: 1) Encapsulating plant protein anti-breast cancer bioactive peptides using polysaccharide-based, carbon-based, metal-based, protein-based, or liposome-based nanoparticles to improve their resistance to gastrointestinal digestive enzymes. 2) Utilizing enzymatic hydrolysis or microbial fermentation methods instead of chemical hydrolysis or thermal hydrolysis, which can not only reduce the generation of by-products and harmful chemical residues but also enhance the activity of plant protein anti-breast cancer peptides. 3) Optimizing the processing conditions of commercial proteases and fermentation technologies to avoid extreme acidic or alkaline pH, which is beneficial for the stability and bioactivity of plant protein-derived bioactive peptides. 4) When processing plant protein bioactive peptides into functional foods or incorporating them as ingredients to functional foods, it is advisable to avoid incorporation into overly acidic beverages, as this may lead to loss of anti-breast cancer activity.

### 4.3 Application of plant protein-derived anti-breast cancer peptides

#### 4.3.1 Medical applications

Active peptides (e.g., Arg-Gly-Asp (RGD) peptide, CRGDKGPDC, polypeptides) have been utilized to selectively deliver drugs to receptor-upregulated breast cancer cells by forming peptide-drug-carrier conjugates (Montet et al., 2006; Zhang et al., 2020; Chen et al., 2022). The preparation of peptide-drug-carrier conjugates using active peptides generally requires several criteria. First, the active peptides should possess the ability to selectively bind to cell surface receptors on breast cancer cells (Majumdar and Siahaan, 2012). Second, the receptors must be expressed exclusively on breast cancer cells, or their expression in breast cancer cells should exceed that in non-target cells, enabling specific binding to breast cancer cells. Third, the peptide carriers need to possess sufficient stability in systemic circulation to ensure that they reach effective concentrations in breast cancer cells. Fourth, selecting conjugation sites on the peptide is crucial for maintaining its binding characteristics with the receptor, as drug conjugation may impose steric hindrance that interferes with receptor recognition. The unique advantages of active peptides include ease of modification, high specificity, scalability, stability, biocompatibility, safety, and low immunogenicity; however, they may also pose risks of additional toxicity (Majumdar and Siahaan, 2012; Araste et al., 2018). To date, over 100 active peptide sequences have been identified that can serve as drug delivery vectors, molecules, and cargos, with lengths ranging from 5 to 40 amino acids (Araste et al., 2018). As noted in Chen et al. (2022)’s research, iRGD-PSS@PBAE@JQ1/ORI NPs utilized the ability of the iRGD peptide to specifically bind to integrin receptors αvβ3 and αvβ5, which are highly expressed on breast cancer cell surfaces, thus significantly enhancing the active tumor-targeting and penetration capabilities of the nanoparticles, ultimately leading to improved nanoparticle internalization and therapeutic efficacy. Consequently, compared to other treatment groups, the iRGD-PSS@PBAE@JQ1/ORI NPs group exhibited the highest *in vitro* and *in vivo* anti-breast cancer efficacy. However, no anti-breast cancer drug-peptide conjugate has successfully entered the market, primarily due to the challenges in developing suitable ligands for the targeted receptors on breast cancer cell surfaces and the insufficient understanding of intracellular ligand uptake, action mechanisms, and the pharmacokinetics profiles of drug-peptide conjugates (Majumdar and Siahaan, 2012).

Plant protein-derived anti-breast cancer peptides can also exert therapeutic effects *in vivo*. These therapeutic peptides have several notable advantages, such as small size, low cost, ease of synthesis, ease of modification, rapid absorption, ability to penetrate cell membranes, high anti-cancer activity, and biological and chemical diversity (Caboche et al., 2010; Zhang et al., 2012; Stalmans et al., 2013; Miner-Williams et al., 2014; Ramsey and Flynn, 2015; Wei et al., 2015). Among these, oral therapy of therapeutic peptides offers better safety and enhances patient compliance, while their small size allows them to penetrate cell membranes to target intracellular molecules (Wang L. et al., 2022). Furthermore, PDBP do not accumulate in specific organs (e.g., kidneys or liver), thereby alleviating the metabolic burden on these organs. However, the limitations of plant protein therapeutic peptides are also evident, including susceptibility to degradation by digestive enzymes and serum proteases, poor solubility, and short half-lives (Majumdar and Siahaan, 2012). Currently, researchers primarily focus on the therapeutic activities of anti-breast cancer peptides identified from plant proteins, such as anti-proliferative, anti-metastatic, anti-invasive, and pro-apoptotic effects. This emphasis hinders the development of these plant-based breast cancer therapeutic peptides into clinical treatment agents, as they require further evaluation for stability, toxicity, and clinical trials.

In addition to targeting breast cancer tissues or exerting anti-breast cancer therapeutic effects, PDBP can offer additional health benefits to breast cancer patients. PDBP or plant protein peptide mixtures can be rapidly digested and absorbed by the human body, thereby improving overall health and preventing chronic diseases (Cicero et al., 2017). Notable health benefits include antioxidant, anti-inflammatory, immune-enhancing, anti-fatigue effects, improved digestive health, regulation of lipid metabolism, and promotion of muscle growth and repair, all of which contribute to the recovery of cancer patients and enhance their prognosis (An et al., 2020; Fernández-Tomé and Hernández-Ledesma, 2020; Yuan et al., 2021; Fang et al., 2022; Lin et al., 2022; Zhang D. J. et al., 2023; Di and Jia, 2024). The preventive effects against chronic diseases encompass the prevention of atherosclerosis, tumors, hypertension, diabetes, obesity, and neurodegenerative diseases, assisting frail cancer patients in avoiding additional health issues, thereby improving their life quality.

#### 4.3.2 Industrial applications

Since oxytocin entered the market in 1962, the demand for peptide-based pharmaceuticals has experienced exponential growth over the past 7 years, leading to a significant expansion of the commercial peptide drug market (Pennington et al., 2021). With more than 70 peptide active pharmaceutical ingredients approved and sold worldwide, the necessary manufacturing capabilities to support these products have been established either within the pharmaceutical companies themselves or through contract development and manufacturing organizations (Pennington et al., 2021).

After identifying the amino acid sequences of anti-breast cancer bioactive peptides derived from plant proteins, chemical synthesis can be employed for the large-scale production of these peptides, primarily through solution-phase synthesis and solid-phase synthesis (Masui and Fuse, 2022). The former method offers cost advantages but has the drawback of requiring the removal of intermediate by-products during peptide production to enhance product purity, making the process time-consuming and complex (Jaradat, 2018; Lawrenson, 2018). Moreover, this method is typically suitable for producing bioactive peptides containing 3 to 20 amino acids, as longer peptides exhibit low solubility in organic solvents, resulting in substantial chemical waste during production (Akbarian et al., 2022). With advancements in chemical synthesis techniques, solid-phase synthesis has increasingly become the preferred method for peptide synthesis in the market. This technique relies on the reaction of amino acids that remain shielded by protective chemical groups and unreacted on the resin in the presence of insoluble substances (Alzaydi et al., 2023). Specifically, the fluorenylmethoxycarbonyl group serves as a protective coating for the side chains of the amino acids. The first amino acid is linked to the resin bed via its carboxyl terminus, while its amino terminus and side chain are safeguarded (Espitia et al., 2012; Alzaydi et al., 2023). Following the coupling process, the protective group is cleaved from the amino terminus, making it ready to react with the subsequent amino acid. Coupling reagents facilitate the joining of amino acids, resulting in the progressive extension of the peptide chain (Akbarian et al., 2022). The advantages of this method include simplicity, convenience, and cost-effectiveness, with minimal accumulation of intermediate by-products. However, its drawbacks are also significant, as it requires a large amount of materials to initiate the synthesis process and expensive equipment, resulting in relatively high costs for producing bioactive peptides (Gracia et al., 2009; Akbarian et al., 2022). After the chemical synthesis of bioactive peptides, purification can be achieved through RP-HPLC or ion-exchange chromatography, which removes impurities generated during the synthesis process, such as racemized species, deletions, insertions, and incompletely removed protecting groups (Hühmer et al., 1997; Pennington et al., 2021). Commercial preparative HPLC systems utilize high-flow water, acetonitrile, methanol, or isopropanol solvents to elute products from the chromatography column during purification and salt exchange processes. Subsequently, freeze-drying equipment is employed to sublime organic solvents and water from the purification or reconstitution buffer, yielding an amorphous, fluffy powder (Pardeshi et al., 2023). These synthesized bioactive peptides can then be marketed in large quantities as food additives, nutritional supplements, and pharmaceuticals (de Castro and Sato, 2015; Wen et al., 2020).

## 5 The action mechanism of plant protein-derived anti-breast cancer peptides

**FIGURE 3:**
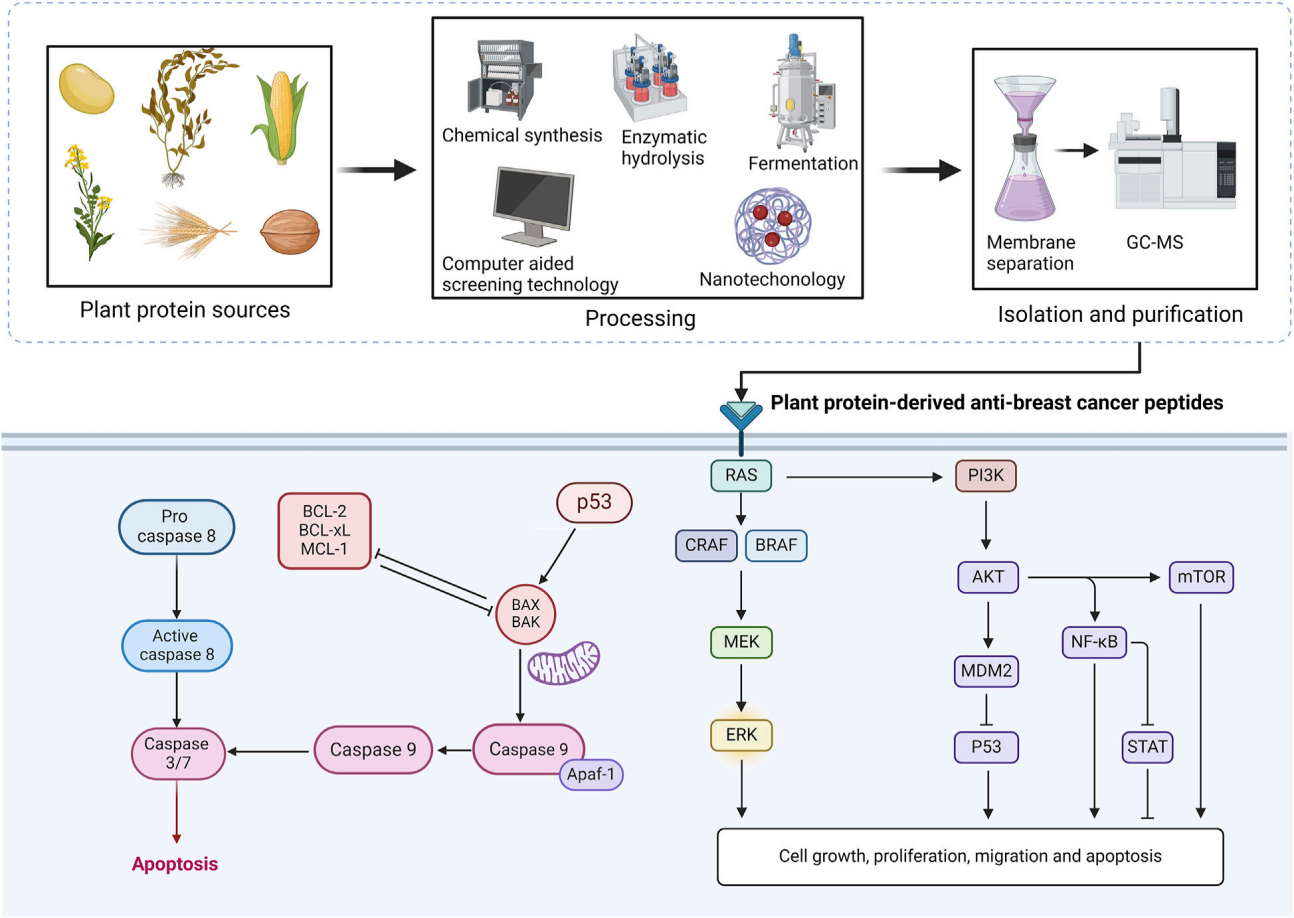
Sources, extraction, isolation, purification of plant protein active peptides and their regulatory mechanisms in breast cancer. BCL-2, B cell lymphoma-2; BCL-xL, B-cell lymphoma-extra-large; MCL-1, myeloid cell leukaemia-1; BAX, BCL-2-associated X; BAK, BCL-2 antagonist killer; MEK, mitogen protein kinase kinase 1 and 2; ERK, extracellular signal-regulated kinase 1/2; AKT1, protein kinase B; PI3K-Akt, phosphoinositide 3-kinase/Akt; protein kinase B; MDM2, murine double minute 2; p53, tumor protein p53; NF-κB, Nuclear factor kappa B; STAT, signal transducers and activators of transcription; mTOR, mammalian target of rapamycin.

The anticancer mechanisms of plant protein-derived anticancer peptides are complex and diverse, and the most important signaling pathways include the p53 signaling pathway, the mitochondrial apoptosis signaling pathway, etc., which are summarized below (Figure 3).

### 5.1 p53 pathway

The p53 protein is a transcription factor known as the “guardian of the genome” because of its critical function in maintaining genomic integrity and is one of the most thoroughly studied tumor suppressor factors to date (Feroz and Sheikh, 2020; Huang, 2021). The p53 gene undergoes mutations in about half of all human malignancies, such as lung cancer, gastric cancer, breast cancer, colorectal cancer, prostate cancer, and skin malignancies (Marei et al., 2021). Once DNA damage happens, the p53 gene on human chromosome 17 undergoes cell cycle arrest. However, if the p53 protein is mutated, the aforementioned cell cycle arrest disappears, allowing damaged DNA to be replicated indefinitely, leading to abnormal cell proliferation and cancer development (Marei et al., 2021).

p53 effectively regulates processes such as apoptosis of cancer cells, oncogene activation, DNA damage, cell cycle arrest, hypoxia, and nutrient deprivation, all of which are closely related to cancer progression (Helton and Chen, 2007; Lützkendorf et al., 2017). The activity of p53 is finely tuned through post-translational modifications, protein-protein interactions, and protein stability (Matheu et al., 2008). In the process of p53 functioning, MDM2 closely regulates the p53 molecule, primarily by ubiquitination and proteasomal degradation to inhibit the activation of p53 (Candeias et al., 2008). Specifically, transcriptionally activated MDM2 by p53 leads to the phosphorylated MDM2 translocating to the nucleus and binding with p53, causing p53 degradation and limiting p53 to a low concentration (Matheu et al., 2008). However, in a stressful environment, when DNA damage happens, ATM/ATR and other kinases cause phosphorylation of p53 and MDM2, resulting in the inhibition of the interaction between p53 and MDM2, thereby stabilizing p53 (Khosravi et al., 1999; Chen et al., 2005; Sun et al., 2017). Besides, Arf also plays an important role in the stabilization of p53. Under normal conditions, Arf is expressed at low levels, but when stress conditions occur and oncogenes are introduced into normal cells, it induces high-level transcription of Arf, leading to the activation of p53, further inhibiting tumor progression (Matheu et al., 2008). In humans, mechanisms such as Arf and DNA damage signaling regulate the activation of p53 signaling together, making the process of p53 signaling function even more complex.

Some studies have explored the activating effects of PDBP on the p53 signaling pathway, allowing them to play a role in impeding cancer progression. The bioactive peptide RQSHFANAOP extracted from chickpea protein has been shown to induce high expression of p53 protein in breast cancer cells (Xue et al., 2015). Additionally, molecular docking results have shown that RQSHFANAOP forms a strong bond with the p53 protein (PDB: 4HJE) through hydrogen bonds, providing dual evidence that chickpea peptides produce anticancer effects by activating the p53 signaling pathway. However, the exploration of this study regarding the p53 signaling pathway is insufficiently robust, lacking strong evidence like western blotting and immunofluorescence to demonstrate the activation of the p53 signal in breast cancer cells. Similarly, Shi et al. (2022) have isolated peptides with molecular weights less than 3 kDa from *B. javanica* proteins. These mixed peptides not only reduce the viability of breast cancer cells to 50% at concentrations as low as 0.25 μg/mL but also significantly increase the proportion of apoptotic cells and block cell cycle progression. Shi et al. (2022) also explored the mechanisms by which *B. javanica* peptides act, and results showed that they are associated with the activation of the p53 signaling pathway. However, further research is needed to explore how different plant protein sources of anti-breast cancer peptides mediate the p53 pathway. In addition, the activation of upstream and downstream molecules of the p53 signaling pathway by plant-derived peptides needs to be further explored to reveal the details of their action fully.

### 5.2 Caspase-and mitochondrial-dependent pathway

Compared to healthy cells, cancer cells have higher metabolic demands and antioxidant defenses (Liang et al., 2013). Cancer cells primarily utilize two main energy supply channels: oxidative glycolysis and oxidative phosphorylation (OXPHOS) and they are tightly coupled and competitively interact (Zheng, 2012). The entire glycolysis process occurs in the cytoplasm, causing the release of two ATP and pyruvate, the latter serving as fuel for OXPHOS. In aerobic conditions, pyruvate enters the mitochondria and initiates the tricarboxylic acid cycle and OXPHOS process, releasing 36 ATP (Zheng, 2012). Under anaerobic conditions, pyruvate undergoes a reduction reaction in the cytoplasm to form lactate, which is then transported out of the cell by monocarboxylate transporters on the cell membrane (Wang X. C. et al., 2022). Proportionally, glycolysis contributes 1%–64% of the ATP in cancer cells, which quickly meets the energy needs of cancer cells. Many cancers also use OXPHOS as a primary energy supply channel; for example, in breast cancer MCF-7 cells, OXPHOS contributes 91% of ATP in aerobic environments and 36% of ATP in hypoxic environments (Zheng, 2012). Therefore, mitochondria play a crucial role in cancer tissue growth and metabolism, and targeting mitochondria and disrupting the OXPHOS mechanism are regarded as effective cancer treatment methods in many studies.

One way to regulate oncogenic and tumor-suppressing signaling pathways is by modulating the sensitivity of cells to mitochondrial-dependent apoptosis through the convergence of the Bcl-2 family of pro-apoptotic and anti-apoptotic proteins, thereby activating or inhibiting cancer-promoting and cancer-suppressing pathways (Trotta and Chipuk, 2017). When subjected to stress or damage, such as nutrient deficiency or DNA damage, the mitochondrial-dependent or intrinsic pathway of apoptosis is activated (Ricci et al., 2008). This pathway interacts with the pro-apoptotic members of the Bcl-2 family, such as BAK, namely, Bcl-2 antagonist killer 1, and BAX, namely, Bcl-2-associated X protein, which together facilitate the formation of pores in the outer mitochondrial membrane (OMM) (Trotta and Chipuk, 2017). This process, also known as mitochondrial outer membrane permeabilization, leads to the release of pro-apoptotic factors, such as cytochrome c, which in turn interacts with APAF-1, ultimately triggering the recruitment and activation of cysteine-aspartic proteases (caspases) (Creagh et al., 2003). Specifically, once caspase-9 is initiated, it propagates the death signal by activating downstream caspases (Creagh et al., 2003). Caspase-3 and caspase-7 are simultaneously activated by the caspase-9 signal within the apoptosome, and the activated caspase-3 then activates caspase-2 and caspase-6, subsequently activating caspase-8 and caspase-10 in a caspase-6 dependent manner (Creagh et al., 2003; Brentnall et al., 2013; Yan et al., 2018). Caspase-dependent cleavage of a large number of substrates marks the final stage of apoptosis, leading to the effective packaging and elimination of the target cell.

Some PDBP have been shown to regulate the caspase-dependent apoptosis pathway and mitochondrial-dependent apoptosis pathway, participating in promoting apoptosis in breast cancer. Lam and Ng (2011) found that ANDISFNFVRFNETNLILGG at a concentration of only 0.2 μM triggered a 50% inhibition rate in MCF-7 cells, leading to membrane depolarization and cell cycle arrest at the G2/M phase. They interpreted these changes as activation of the mitochondrial apoptosis signaling pathway. Similarly, the lunasin peptide in soybean has been shown to inhibit breast cancer progression, effectively increasing the expression of caspase-3, caspase-7, caspase-14, and elevating the Bax/Bcl-2 level, indicating that lunasin can activate both the caspase- and mitochondrial-dependent pathways (Hao et al., 2022). Wang et al. (2016) found that the peptide extracted from rapeseed protein with a sequence of WYP caused a significant decline in the vitality of MCF-7 cells, which was associated with the activation of the mitochondrial apoptosis signaling pathway, as evidenced by increased expression of p53 and Bax proteins and decreased expression of Bcl-2 protein. Studies by Liao et al. (2016), Li et al. (2014a), and Li et al. (2014b) also observed similar views, demonstrating the close relationship between breast cancer cell apoptosis and the caspase- and mitochondrial-dependent pathways, as well as the regulatory effects of PDBP on them. It is now possible to identify additional anti-breast cancer bioactive peptides from other protein sources, such as pea proteins, sesame proteins, hazelnut proteins, and peanut proteins, and to explore whether these active peptides act via the caspase-and mitochondrial-dependent pathway.

### 5.3 PI3K-Akt pathway

The PI3K-Akt signaling pathway, composed of PI3K and PKB, is one of the crucial pathways whose regulation is associated with various human cancers (Miricescu et al., 2020). Abnormalities in the PI3K/AKT pathway are closely related to tumor occurrence, proliferation, apoptosis, invasion, metabolism, metastasis, angiogenesis, epithelial-mesenchymal transition, stem cell-like phenotype, immune microenvironment, and drug resistance in cancer cells (Jiang et al., 2020). This signaling pathway is sensitive to oncogenes, growth factor receptors, cytokines, and hormones, such as insulin receptor tyrosine kinase, insulin and epidermal growth factor receptor and can be activated by them (Noorolyai et al., 2019). Additionally, PI3K can be directly or indirectly activated by small Ras-related GTPases, whose interaction is essential for maintaining the survival and proliferation of cancer cells (Rascio et al., 2021).

Several studies indicate that the PI3K pathway is activated through the downregulation or loss of PTEN, an essential tumor suppressor protein that encodes a phosphatidylinositol-3,4,5-trisphosphate (Noorolyai et al., 2019; Rascio et al., 2021). Once activated, PI3K acts on its downstream effector serine/threonine kinase Akt and then activated Akt translocates from the cell membrane to nucleus, phosphorylating and triggering downstream effector molecules (Matheny and Adamo, 2009; Liao and Hung, 2010). Akt phosphorylation contributes to the progression of cancer through suppressing transcription factors members of the FOXO family, which are responsible for suppressing cancer growth and proliferation (Rascio et al., 2021).

A primary target of PI3K/Akt is the serine/threonine kinase mTOR (Morgensztern and McLeod, 2005). Akt causes the phosphorylation and inhibition of the TSC2 protein, thereby suppressing Ras homolog and leading to the activation of mTORC1, which plays a crucial role in the process of cell proliferation, protein synthesis, and energy storage as a downstream molecule (Hassan et al., 2013; Rascio et al., 2021). Phosphorylation of mTOR is considered a marker of tumour progression and mediates the activation of downstream targets such as S6K and 4E-BP1, the activation of which leads to an increased risk of tumor development and poor prognosis (Rascio et al., 2021).

Recently, drugs derived from natural products have attracted significant interest. It is estimated that at least 1/3 of the top twenty commercially available drugs are derived from natural sources (Tewari et al., 2022). *Porphyra haitanesis* is an important source of anticancer peptides, from which active peptides have been successfully isolated and their effects on the PI3K/Akt signaling have been extensively investigated. Park et al. (2014) found that *P. haitanesis* oligopeptide (KKAAE) inhibited proliferation and promoted apoptosis of breast cancer cells, which was attributed to the activation of the PI3K-Akt signaling pathway. However, there is limited research on PDBP regulating breast cancer progression through the PI3K-Akt signaling pathway.

### 5.4 Other signaling pathways

In addition to the aforementioned mechanisms, PDBP also regulate the progression of breast cancer through modulation of the NF-κB signaling pathways. Jiang et al. (2016) have reported that in MCF-7 and MDA-MB-231 cell models, the use of lunasin can inhibit activity, migration, motility, and invasion of breast cancer cells, and decrease the activity and expression of MMP2 and MMP9, which is attributed to the inhibition of NF-κB signaling pathways. Furthermore, PDBP are also considered to exhibit immunomodulatory activities in breast cancer model. As demonstrated by Ma et al. (2015), a peptide derived from walnut hydrolysate, with the sequence CTLEW, was proved to act by stimulating spleen lymphocytes and macrophages. In this study, CTLEW significantly increased the production of IL-2 in spleen lymphocytes and enhanced the phagocytic activity and NO accumulation of macrophage. Additionally, PDBP have been shown to exert anti-breast cancer effects through pathways like ER signaling and IGF signaling pathway, although related research is still scarce (Park et al., 2014; Hsieh et al., 2023). The pathogenesis of breast cancer is very complex, and PDBP often exerts inhibitory effects by modulating multiple signaling pathways, but a lot remains to be done before this field is fully understood.

## 6 Nanoparticles loaded with anti-breast cancer peptides

PDBP face multiple challenges during digestion and absorption in the body, such as stomach proteases, pancreatic proteases, stomach acid, and mucus (Zhang Z. Q. et al., 2023). Additionally, these peptides may have issues with stability, solubility, and light instability, which limit their storage, absorption, and functionality in the body (Luo et al., 2023). Degraded PDBP often lose their original high bioactivity, leading to lower absorption rates and uncertain therapeutic effects, thus reducing the practical value of these peptides (Horner et al., 2016; Gong et al., 2022).

Nanoparticles are artificially synthesized particles ranging in size from 1 to 1000 nm, and they are known for enhancing drug absorption efficiency and providing flexible administration methods, such as via nasal, vaginal, oral, inhalation, and skin routes (Choi et al., 2014; Shah et al., 2020). Nanoparticles can be categorized into various forms in terms of materials, such as nanoemulsions, nanohydrogels, polymer nanoparticles, ceramic nanoparticles, metal nanoparticles, polysaccharide nanoparticles, and lipid nanoparticles, each of which has a different function (Zhang Y. Z. et al., 2024). Nanoparticles play a crucial role in targeted drug delivery, reducing side effects, lowering dosing frequency, and improving drug utilization, stability, and solubility, which makes them beneficial for various small-molecule drugs like paclitaxel, curcumin, and DOX (Wen et al., 2022; Lee et al., 2023; Lin et al., 2023). Thus, their application is gradually becoming more widespread in fields like medicine, material science, food science, and pharmaceutics.

In the field of food science, protein-based nanocarriers, polysaccharide-based nanocarriers, lipid-based nanocarriers, and hybrid nanocarriers are generally used to encapsulate PDBP, which can significantly enhance their antioxidant, anti-inflammatory, anti-cancer, and anti-diabetic properties (Zhang Z. Q. et al., 2023). This enhancement may be attributed to the effective protection provided by the nanoparticles and/or the synergistic effects between the PDBP and the nanoparticle carriers (Asr et al., 2023; Zhang Z. Q. et al., 2023). Thus, nanoparticles are often more effective in killing breast cancer cells and inhibiting their invasion, migration, and metastasis, ultimately leading to better therapeutic outcomes (Yen et al., 2023; Yu and Zhu, 2024). The regulatory mechanisms may vary depending on the type of PDBP and nanoparticle carrier used.

PDBP may be formulated into nanoparticles via encapsulation and modification, or as protein-based nanoparticles that, upon oral administration, release anti-breast cancer PDBP following gastrointestinal hydrolysis. Ilhan-Ayisigi et al. (2021) employed the ion gelation technique to mix the rice husk protein extract with sodium tripolyphosphate, which was then dripped into a 0.05% (w/v) chitosan solution to yield RHC-NPs. The results showed that the encapsulation efficiency of RHC-NPs was 89%, with the prepared nanoparticles having an average particle size of 256.4 ± 33.4 nm and a PDI of 0.452. The nanoparticles were spherical with a smooth surface and exhibited enhanced thermal stability. Additionally, MTT assays showed that the designed nanoparticles effectively inhibited the survival of MCF7 cells (IC_50_ value of 3.58 μg/mL), and Hoechst 33342 staining indicated that the nanoparticles caused nuclear fragmentation and the formation of apoptotic bodies. In the study of Chen et al. (2022), iRGD-conjugated PSS (iRGD-PSS) was synthesized through amidation reaction between the carboxyl groups of PSS and the terminal amino groups of iRGD in the presence of EDC and NHS. Subsequently, JQ1, ORI, and ssPBAE were mixed in DMSO under stirring for 2 h and then the mixture was dripped into an aqueous solution containing iRGD-PSS and PVP, stirred for 2 h, and dialyzed in deionized water to obtain a dispersion of iRGD-PSS@PBAE@JQ1/ORI nanoparticles. The obtained NPs had a particle size of 162.0 ± 14.9 nm, PDI of 0.142, and zeta potential of −46.6 mV. These nanoparticles demonstrated targeted breast tumor-killing effects, exhibited a lower IC_50_ value (2.5 μg/mL for JQ1, 1.8 μg/mL for ORI and 0.69 μg/mL for iRGD-PSS@PBAE@JQ1/ORI NPs, significantly increased cellular ROS accumulation, induced immune activation, reduced lactate secretion, and inhibited tumor growth and lung metastasis in 4T1 tumor-bearing mice. Specially, ARPI powder was added to DDI H_2_O and magnetically stirred at room temperature for 2 h to prepare ARPI solution (2.0 mg/mL). Subsequently, DOX was added to the chitosan solution (0.1 mg/mL, dissolving chitosan powder in 1% (v/v) acetic acid), and the resulting solution was gradually dripped into an equal volume of the ARPI solution, ultimately yielding DOX-ARPI/CS NPs (Wang et al., 2018). The designed DOX-ARPI/CS NPs were orange-colored, spherical, with an average diameter of 188 nm, and a zeta potential of −11 mV and were capable of releasing DOX and pro-apoptotic peptides (Ala-Gly-Ser, Pro-Ala-Ser, and Tyr-Thr) under acidic conditions. DOX-ARPI/CS NPs activated the mitochondrial-dependent apoptosis pathway (upregulating expressions of p53, Bax, and pro-caspase-3 and downregulating expressions of Bcl-2), enhanced the inhibition effects on MDA-MB-231 cells (IC_50_: DOX-ARPI/CS NPs vs. DOX: 0.5 ± 0.08 μM vs. 1.07 ± 0.10 μM) and the ratio of the monomeric form of membrane-permeant JC-1 dye, which was assigned to DOX-ARPI/CS NPs inducing stronger lysosomal swelling and disruption, promoting greater lysosomal escape and nuclear trafficking of DOX, thereby leading to enhanced apoptosis in cancer cells. Additionally, the anti-breast cancer effect was further validated in an MDA-MB-231 breast tumor mouse model, where mice treated with DOX-ARPI/CS NPs had the smallest tumor volume, highest caspase-3 expression, and longest survival time. This study utilized the advantage of the release of highly active anticancer peptides from rapeseed proteins to synergistically kill tumor cells with DOX, representing an innovative design method for plant protein anti-tumor nanoparticles. Overall, the three types of nanoparticles based on anticancer active peptides have their own advantages, and all of them show greater stability and killing effect against breast cancer compared to anticancer peptides or anticancer drugs alone.

Generally, the higher the concentration of PDBP-loaded nanoparticles, the greater their anti-breast cancer activity. This was confirmed in the study of Turky et al. (2024). The lyophilized peptide NRC-07 (RWGKWFKKATHVGKHVGKAALTAYL) was mixed with sodium tripolyphosphate (TPP) to prepare a P-TPP solution, which was then thoroughly mixed with chitosan solution to self-assemble into P-CS-NPs. The results revealed that the particle size of P-CS-NPs was 153 ± 35 nm, with a zeta potential of +40.6 ± 2.0 mV and an EE of 95%, indicating a non-aggregated state with a uniform size distribution. As the concentration of nanoparticles increased from 10 μM to 150 μM, the viability of MDA-MB-231 cells decreased from approximately 85% to 52.5% ± 5.0%, while the viability of MCF-7 cells decreased from approximately 85% to 14.0% ± 3.0%, suggesting that MCF-7 cells are more sensitive to NRC-07 treatment than MDA-MB-231 cells. However, this study did not assess whether high concentrations of nanoparticles could have toxic effects on healthy human cells. Similar observations were obtained by Chen et al. (2022), who stated that as the concentration of iRGD-PSS@PBAE@JQ1/ORI NPs increased from 0.1 μg/mL to 4.0 μg/mL, the viability of cancer cells gradually decreased from around 95%–30%. The tumor-bearing mouse experiments indicated that high concentrations of iRGD-PSS@PBAE@JQ1/ORI NPs (5 mg/kg) not only did not impact the body weight of the mice but also exhibited significant accumulation in the tumors, with minimal distribution in the lungs, kidneys, and liver. This suggests that the use of iRGD-PSS@PBAE@JQ1/ORI NPs does not cause obvious side and toxic effects, and can further reduce the damage to lungs, kidneys, and livers while enhancing the cytotoxic effects on tumor tissues.

Currently, there are many directions to be explored in the study of nanoparticle embedding of PDBP, such as optimizing nanoparticle formulations, enhancing the embedding capacity of plant protein-derived anti-breast cancer peptides, and improving drug efficacy. Furthermore, using plant proteins as nanoparticle carriers, modifying nanoparticle carriers with plant protein-derived peptides, or using plant protein-derived peptides as encapsulated drugs are all research directions worth exploring and further developing. Lastly, the reasons for the enhanced tumor-killing effects of PDBP nanoparticles require further investigation. Although these plant protein peptide-based nanoparticles have great prospects and advantages, their application is limited due to challenges involving cutting-edge technology, high production costs, and targeted release of nanoparticles. Addressing these scientific and practical issues will help advance the field of nanoscience and have profound implications for the progress of precision medicine.

## 7 Conclusion, limitations, and future perspectives

PDBP have shown notable advantages in the treatment of breast cancer, such as low cost, low side effects, and high compliance, which have garnered widespread attention and recognition among scholars. Anti-breast cancer peptides from various plant protein sources (legume proteins, grain proteins, marine proteins, and oilseed proteins) can inhibit breast cancer proliferation, migration, and invasion, and promote apoptosis through various mechanisms, such as the p53 pathway, caspase-and mitochondrial-dependent pathway, PI3K-Akt pathway, and NF-κB pathway. PDBP can be assembled into small-size nanoparticles with other nanomaterials through modification or encapsulation. This approach further enhances the stability, targeting capacity, and tumor-killing effects of plant protein-derived anti-breast cancer peptides, enabling them to more effectively inhibit breast cancer progression and exhibit broader applications in the fields of food, materials, and medicine.

However, this review has the following limitations:

1. Research on certain plant protein-derived anti-breast cancer peptides, such as those from tomato protein, sunflower protein, potato protein, spinach protein, and red bean, are very limited, leading to the present review focusing on the summary of commonly known plant protein anti-cancer peptides. Additionally, studies related to the mechanisms of PDBP in breast cancer are relatively few, especially those involving the PI3K-Akt and p38/JNK/ERK pathways, making the section on the action mechanisms of plant peptides less comprehensive.
2. Due to a lack of relevant studies, this review does not summarize clinical research on the use of PDBP in treating breast cancer.

Plant protein-derived anti-breast cancer peptides represent a promising research direction. The following unresolved issues will be the future focus of scholars:

1. Glycopeptides, lipid peptides, and selenium-rich peptides may exhibit stronger anti-breast cancer effects and nutritional value, and the extraction of these types of active peptides from plants and the detection of their anti-breast cancer activity is also a valuable research direction.
2. Most plant protein-derived anti-breast cancer peptides are obtained through enzymatic hydrolysis and fermentation. However, even under the same conditions to hydrolyze the same amount of protein can produce different peptide mixtures, leading to instability in the fermentation or enzymatic products. Controlling fermentation or enzymatic conditions to obtain stable peptide mixtures will facilitate the clinical application of plant protein-derived anti-breast cancer peptides.
3. New plant proteins, such as peanut protein, flaxseed protein, pumpkin seed protein, sunflower seed protein, and potato protein, may contain peptides with higher anti-breast cancer activity, and these plant proteins can be enzymatically digested or microbiologically processed to release anti-breast cancer active peptides, and their sequences and anti-breast cancer activities are worthy of study.
4. The anti-proliferative, metastatic, invasive and pro-apoptotic activities of PDBP against breast cancer, its relationship with its molecular weight, secondary structure and amino acid composition needs to be further investigated.

From the food industry’s perspective, understanding the anti-breast cancer effects and mechanisms of PDBP will provide guidance for the development of plant-derived protein peptides into pharmaceutical formulations, functional foods, and dietary supplements. Furthermore, this review also contributes to increasing the added value of plant proteins and offers support for adjunctive treatments of patients with breast cancer. In the medical field, the isolation of plant protein-derived anti-breast cancer peptides meets patients’ needs for effective natural medicines with few side effects. These medicines have lower production costs compared to common anti-cancer drugs like paclitaxel, potentially making them effective alternatives or allowing them to be used in conjunction with chemotherapeutic drugs to improve their effectiveness while reducing their toxicity. This review provides guidance for the future development of plant-derived peptides with higher anti-cancer activity, and helps to promote the use of PDBP in the clinical field.

## Glossary

4E-BP1: 4E binding protein 1
ACE: Angiotensin I-converting enzyme
APAF-1: Apoptotic protease activating factor 1
ARPI: acetylated rapeseed protein isolate
ATM/ATR: Ataxia-telangiectasia mutated/ataxia-telangiectasia and Rad3-related
Bcl-2: B cell lymphoma-2
BET: extra-terminal
CNKI: Chinese China National Knowledge Infrastructure
DOX: doxorubicin
EDC: 1-(3-dimethylaminoprophyl)-3-ethylcarbodiimide hydrochloride
FAK/Akt/ERK: Focal adhesion kinase/Akt/extracellular signal-regulated kinase
FOX: Forkhead box O
IGF: Insulin-like growth factor
iRGD: CRGDKGPDC
JQ1: BET inhibitor.
MDM2: Murine double minute 2
MMP2: Matrix metalloproteinases 2
mTOR: mammalian target of rapamycin.
NF-κB: Nuclear factor kappa B.
NHS: N-hydroxysuccinimide.
ORI: oridonin
PDBP: Plant protein-derived bioactive peptides.
PDI: polydispersity index.
PI3K-Akt: Phosphoinositide 3-kinase/Akt.
PKB: Serine/threonine-protein kinase B.
PSS: propylene glycol alginate sodium.
RHC-NPs: rice husk protein hydrolysates-encapsulated chitosan-based nanoparticles.
ROS: reactive oxygen species.
S6K: S6kinase
ssPBAE: disulfide-containing poly (β-amino ester)
TSC2: tuberous sclerosis complex 2

## References

[B1] Agrawal K. Shahani L. J. B. E. P. L. S. (2021). Pumpkin seeds and oil as sources of bioactive compounds and their therapeutic uses. A Rev. 10, 01–08.

[B2] Ahmed S. Mirzaei H. Aschner M. Khan A. Al-Harrasi A. Khan H. (2021). Marine peptides in breast cancer: therapeutic and mechanistic understanding. Biomed. and Pharmacother. 142, 112038. 10.1016/j.biopha.2021.112038 34411915

[B3] Ai Y. F. Jane J. L. (2016). Macronutrients in corn and human nutrition. Compr. Rev. Food Sci. Food Saf. 15 (3), 581–598. 10.1111/1541-4337.12192 33401819

[B4] Akbarian M. Khani A. Eghbalpour S. Uversky V. N. (2022). Bioactive peptides: synthesis, sources, applications, and proposed mechanisms of action. Int. J. Mol. Sci. 23 (3), 1445. 10.3390/ijms23031445 35163367 PMC8836030

[B5] Alzaydi A. Barbhuiya R. I. Routray W. Elsayed A. Singh A. (2023). Bioactive peptides: synthesis, applications, and associated challenges. Food Bioeng. 2 (3), 273–290. 10.1002/fbe2.12057

[B6] Amagliani L. O'Regan J. Kelly A. L. O'Mahony J. A. (2017). The composition, extraction, functionality and applications of rice proteins: a review. Trends Food Sci. and Technol. 64, 1–12. 10.1016/j.tifs.2017.01.008

[B7] An J. Feng Y. X. Zheng J. H. Addy M. Zhang L. Ren D. F. (2020). The immune-enhancing potential of peptide fractions from fermented *Spirulina platensis* by mixed probiotics. J. Food Biochem. 44 (7), e13245. 10.1111/jfbc.13245 32462664

[B8] Anand P. Kunnumakara A. B. Sundaram C. Harikumar K. B. Tharakan S. T. Lai O. S. (2008). Cancer is a preventable disease that requires major lifestyle changes. Pharm. Res. 25 (9), 2097–2116. 10.1007/s11095-008-9661-9 18626751 PMC2515569

[B9] Anjum F. M. Nadeem M. Khan M. I. Hussain S. (2012). Nutritional and therapeutic potential of sunflower seeds: a review. Br. Food J. 114 (4-5), 544–552. 10.1108/00070701211219559

[B10] Araste F. Abnous K. Hashemi M. Taghdisi S. M. Ramezani M. Alibolandi M. (2018). Peptide-based targeted therapeutics: focus on cancer treatment. J. Control. Release 292, 141–162. 10.1016/j.jconrel.2018.11.004 30408554

[B11] Asr M. H. Dayani F. Segherloo F. S. Kamedi A. Neill A. O. MacLoughlin R. (2023). Lipid nanoparticles as promising carriers for mRNA vaccines for viral lung infections. Pharmaceutics 15 (4), 1127. 10.3390/pharmaceutics15041127 37111613 PMC10146241

[B12] Avilés-Gaxiola S. Gutiérrez-Grijalva E. P. León-Felix J. Angulo-Escalante M. A. Heredia J. B. (2020). Peptides in colorectal cancer: current state of Knowledge. Plant Foods Hum. Nutr. 75 (4), 467–476. 10.1007/s11130-020-00856-6 32964320

[B13] Bangar S. P. Suri S. Malakar S. Sharma N. Whiteside W. S. (2022). Influence of processing techniques on the protein quality of major and minor millet crops: a review. J. Food Process. Preserv. 46 (12). 10.1111/jfpp.17042

[B14] Bekhit A. E. A. Shavandi A. Jodjaja T. Birch J. Teh S. Ahmed I. A. M. (2018). Flaxseed: composition, detoxification, utilization, and opportunities. Biocatal. Agric. Biotechnol. 13, 129–152. 10.1016/j.bcab.2017.11.017

[B15] Bhadkaria A. Narvekar D. T. Kanekar S. Devasya R. P. Srivastava N. Bhagyawant S. S. (2023). Peptide fraction from moth bean *(Vigna aconitifolia* (Jacq.)) seed protein hydrolysate demonstrates multifunctional characteristics. Process Biochem. 134, 165–174. 10.1016/j.procbio.2023.09.026

[B16] Boopathy N. S. Kathiresan K. (2010). Anticancer drugs from marine flora: an overview. J. Oncol. 2010, 214186. 10.1155/2010/214186 21461373 PMC3065217

[B17] Brentnall M. Rodriguez-Menocal L. De Guevara R. L. Cepero E. Boise L. H. (2013). Caspase-9, caspase-3 and caspase-7 have distinct roles during intrinsic apoptosis. Bmc Cell. Biol. 14, 32. 10.1186/1471-2121-14-32 23834359 PMC3710246

[B18] Burton R. A. Andres M. Cole M. Cowley J. M. Augustin M. A. (2022). Industrial hemp seed: from the field to value-added food ingredients. J. Cannabis Res. 4 (1), 45. 10.1186/s42238-022-00156-7 35906681 PMC9338676

[B19] Caboche S. Leclère V. Pupin M. Kucherov G. Jacques P. (2010). Diversity of monomers in nonribosomal peptides: towards the prediction of origin and biological activity. J. Bacteriol. 192 (19), 5143–5150. 10.1128/jb.00315-10 20693331 PMC2944527

[B20] Candeias M. M. Malbert-Colas L. Powell D. J. Daskalogianni C. Maslon M. M. Naski N. (2008). *p53* mrNA controls p53 activity by managing Mdm2 functions. Nat. Cell. Biol. 10 (9), 1098–1105. 10.1038/ncb1770 19160491

[B21] Cao J. Wang J. P. Wang S. C. Xu X. M. (2016). Porphyra species: a mini-review of its pharmacological and nutritional properties. J. Med. Food 19 (2), 111–119. 10.1089/jmf.2015.3426 26653974

[B22] Carbonaro M. Nucara A. (2022). Legume proteins and peptides as compounds in nutraceuticals: a structural basis for dietary health effects. Nutrients 14 (6), 1188. 10.3390/nu14061188 35334845 PMC8955165

[B23] Cellarier E. Durando X. Vasson M. P. Farges M. C. Demiden A. Maurizis J. C. (2003). Methionine dependency and cancer treatment. Cancer Treat. Rev. 29 (6), 489–499. 10.1016/s0305-7372(03)00118-x 14585259

[B24] Chai K. F. Voo A. Y. H. Chen W. N. (2020). Bioactive peptides from food fermentation: a comprehensive review of their sources, bioactivities, applications, and future development. Compr. Rev. Food Sci. Food Saf. 19 (6), 3825–3885. 10.1111/1541-4337.12651 33337042

[B25] Chaipoot S. Punfa W. Ounjaijean S. Phongphisutthinant R. Kulprachakarn K. Parklak W. (2023). Antioxidant, anti-diabetic, anti-obesity, and antihypertensive properties of protein hydrolysate and peptide fractions from black sesame cake. Molecules 28 (1), 211. 10.3390/molecules28010211 PMC9821986 36615405

[B26] Chang W. H. Liu J. J. Chen C. H. Huang T. S. Lu F. J. (2002). Growth inhibition and induction of apoptosis in MCF-7 breast cancer cells by fermented soy milk. Nutr. Cancer-an Int. J. 43 (2), 214–226. 10.1207/s15327914nc432_12 12588701

[B27] Chen B. W. Liu X. H. Li Y. N. Shan T. H. Bai L. Y. Li C. Y. (2022). iRGD tumor-penetrating peptide-modified nano-delivery system based on a marine sulfated polysaccharide for enhanced anti-tumor efficiency against breast cancer. Int. J. Nanomedicine 17, 617–633. 10.2147/ijn.S343902 35173433 PMC8842734

[B28] Chen L. H. Gilkes D. M. Pan Y. Lane W. S. Chen J. D. (2005). ATM and Chk2-dependent phosphorylation of MDMX contribute to p53 activation after DNA damage. Embo J. 24 (19), 3411–3422. 10.1038/sj.emboj.7600812 16163388 PMC1276172

[B29] Chen Y. H. Li J. Dong N. G. Zhang Y. Q. Lu X. D. Hao Y. B. (2020). Separation and identification of ACE inhibitory peptides from defatted walnut meal. Eur. Food Res. Technol. 246 (10), 2029–2038. 10.1007/s00217-020-03553-5

[B30] Cheung R. C. F. Ng T. B. Wong J. H. (2015). Marine peptides: bioactivities and applications. Mar. Drugs 13 (7), 4006–4043. 10.3390/md13074006 26132844 PMC4515606

[B31] Chiangjong W. Chutipongtanate S. Hongeng S. (2020). Anticancer peptide: physicochemical property, functional aspect and trend in clinical application (Review). Int. J. Oncol. 57 (3), 678–696. 10.3892/ijo.2020.5099 32705178 PMC7384845

[B32] Chmielewska A. Kozlowska M. Rachwal D. Wnukowski P. Amarowicz R. Nebesny E. (2021). Canola/rapeseed protein - nutritional value, functionality and food application: a review. Crit. Rev. Food Sci. Nutr. 61 (22), 3836–3856. 10.1080/10408398.2020.1809342 32907356

[B33] Choi J. S. Cao J. Naeem M. Noh J. Hasan N. Choi H. K. (2014). Size-controlled biodegradable nanoparticles: preparation and size-dependent cellular uptake and tumor cell growth inhibition. Colloids Surfaces B-Biointerfaces 122, 545–551. 10.1016/j.colsurfb.2014.07.030 25108477

[B34] Cian R. E. Nardo A. E. Garzón A. G. Añon M. C. Drago S. R. (2022). Identification and *in silico* study of a novel dipeptidyl peptidase IV inhibitory peptide derived from green seaweed *Ulva* spp. hydrolysates. Lwt-Food Sci. Technol. 154, 112738. 10.1016/j.lwt.2021.112738

[B35] Cicero A. F. G. Fogacci F. Colletti A. (2017). Potential role of bioactive peptides in prevention and treatment of chronic diseases: a narrative review. Br. J. Pharmacol. 174 (11), 1378–1394. 10.1111/bph.13608 27572703 PMC5429326

[B36] Creagh E. M. Conroy H. Martin S. J. (2003). Caspase-activation pathways in apoptosis and immunity. Immunol. Rev. 193 (1), 10–21. 10.1034/j.1600-065X.2003.00048.x 12752666

[B37] Cruz-Casas D. E. Aguilar C. N. Ascacio-Valdés J. A. Rodríguez-Herrera R. Chávez-González M. L. Flores-Gallegos A. C. (2021). Enzymatic hydrolysis and microbial fermentation: the most favorable biotechnological methods for the release of bioactive peptides. Food Chem. Mol. Sci. 3, 100047. 10.1016/j.fochms.2021.100047 PMC8991988 35415659

[B38] Daliri E. B. M. Oh D. H. Lee B. H. (2017). Bioactive peptides. Foods 6 (5), 32. 10.3390/foods6050032 28445415 PMC5447908

[B39] Da Silva A. C. da Costa Santos D. Junior D. L. T. da Silva P. B. dos Santos R. C. Siviero A. (2018). “Cowpea: a strategic legume species for food security and health,” in Legume seed nutraceutical research. IntechOpen.

[B40] da Silva A. C. de Freitas Barbosa M. da Silva P. B. de Oliveira J. P. da Silva T. L. Junior D. L. T. (2021). Health benefits and industrial applications of functional cowpea seed proteins, 1–12.

[B41] de Castro R. J. S. Sato H. H. (2015). Biologically active peptides: processes for their generation, purification and identification and applications as natural additives in the food and pharmaceutical industries. Food Res. Int. 74, 185–198. 10.1016/j.foodres.2015.05.013 28411983

[B42] de Haan S. Burgos G. Liria R. Rodriguez F. Creed-Kanashiro H. M. Bonierbale M. (2019). The nutritional contribution of potato varietal diversity in andean food systems: a case study. Am. J. Potato Res. 96 (2), 151–163. 10.1007/s12230-018-09707-2

[B43] Dhull S. B. Rani J. Rohilla S. Rose P. K. Chawla P. Kidwai M. K. (2024). Moth bean (*Vigna aconitifolia*) starch: properties, modifications and applications-A review. Legume Sci. 6 (2). 10.1002/leg3.237

[B44] Di C. Jia W. (2024). Food-derived bioactive peptides as momentous food components: can functional peptides passed through the PI3K/Akt/mTOR pathway and NF-κB pathway to repair and protect the skeletal muscle injury? Crit. Rev. Food Sci. Nutr. 64 (25), 9210–9227. 10.1080/10408398.2023.2209192 37171059

[B45] Dotto J. M. Chacha J. S. (2020). The potential of pumpkin seeds as a functional food ingredient: a review. Sci. Afr. 10, e00575. 10.1016/j.sciaf.2020.e00575

[B46] Du Z. Y. Liu J. B. Zhang D. J. Ding L. Wang Y. Z. Tan D. W. (2019). Individual and synergistic antioxidant effects of dipeptides in *in vitro* antioxidant evaluation systems. Int. J. Peptide Res. Ther. 25 (1), 391–399. 10.1007/s10989-018-9684-y

[B47] Espitia P. J. P. Soares N. D. F. Coimbra J. S. D. de Andrade N. J. Cruz R. S. Medeiros E. A. A. (2012). Bioactive peptides: synthesis, properties, and applications in the packaging and preservation of food. Compr. Rev. Food Sci. Food Saf. 11 (2), 187–204. 10.1111/j.1541-4337.2011.00179.x 32368201 PMC7194098

[B48] Fan H. X. Liu H. C. Zhang Y. R. Zhang S. S. Liu T. T. Wang D. W. (2022a). Review on plant-derived bioactive peptides: biological activities, mechanism of action and utilizations in food development. J. Future Foods 2 (2), 143–159. 10.1016/j.jfutfo.2022.03.003

[B49] Fan X. Guo H. M. Teng C. Zhang B. Blecker C. Ren G. X. (2022b). Anti-colon cancer activity of novel peptides isolated from *in vitro* digestion of quinoa protein in caco-2 cells. Foods 11 (2), 194. 10.3390/foods11020194 35053925 PMC8774364

[B50] Fan X. D. Bai L. Mao X. L. Zhang X. W. (2017). Novel peptides with anti-proliferation activity from the *Porphyra haitanesis* hydrolysate. Process Biochem. 60, 98–107. 10.1016/j.procbio.2017.05.018

[B51] Fang E. F. Wong J. H. Ng T. B. (2010). Thermostable Kunitz trypsin inhibitor with cytokine inducing, antitumor and HIV-1 reverse transcriptase inhibitory activities from Korean large black soybeans. J. Biosci. Bioeng. 109 (3), 211–217. 10.1016/j.jbiosc.2009.08.483 20159565

[B52] Fang L. Zhang R. X. Wei Y. Ling K. Lu L. Wang J. (2022). Anti-fatigue effects of fermented soybean protein peptides in mice. J. Sci. Food Agric. 102 (7), 2693–2703. 10.1002/jsfa.11609 34694006

[B53] Fernández-Tomé S. Hernández-Ledesma B. (2020). Gastrointestinal digestion of food proteins under the effects of released bioactive peptides on digestive health. Mol. Nutr. and Food Res. 64 (21), e2000401. 10.1002/mnfr.202000401 32974997

[B54] Feroz W. Sheikh A. M. A. (2020). Exploring the multiple roles of guardian of the genome: P53. Egypt. J. Med. Hum. Genet. 21 (1), 49. 10.1186/s43042-020-00089-x

[B55] Ferrero R. L. Soto-Maldonado C. Weinstein-Oppenheimer C. Cabrera-Muñoz Z. Zúñiga-Hansen M. E. (2021). Antiproliferative rapeseed defatted meal protein and their hydrolysates on MCF-7 breast cancer cells and human fibroblasts. Foods 10 (2), 309. 10.3390/foods10020309 33546198 PMC7913290

[B56] Ferrero R. L. Weinstein-Oppenheimer C. R. Cabrera-Muñoz Z. Zúñiga-Hansen M. E. (2023). The antiproliferative activity of a mixture of peptide and oligosaccharide extracts obtained from defatted rapeseed meal on breast cancer cells and human fibroblasts. Foods 12 (2), 253. 10.3390/foods12020253 36673345 PMC9858037

[B57] Gasymov O. K. Celik S. Agaeva G. Akyuz S. Kecel-Gunduz S. Qocayev N. M. (2021). Evaluation of anti-cancer and *anti*-covid-19 properties of cationic pentapeptide Glu-Gln-Arg-Pro-Arg, from rice bran protein and its D-isomer analogs through molecular docking simulations. J. Mol. Graph. and Model. 108, 107999. 10.1016/j.jmgm.2021.107999 34352727 PMC8325105

[B58] Ghanbari R. Anwar F. Alkharfy K. M. Gilani A. H. Saari N. (2012). Valuable nutrients and functional bioactives in different parts of olive (*Olea europaea* L.)-A review. Int. J. Mol. Sci. 13 (3), 3291–3340. 10.3390/ijms13033291 22489153 PMC3317714

[B59] Gonçalves A. Goufo P. Barros A. Domínguez-Perles R. Trindade H. Rosa E. A. S. (2016). Cowpea (*Vigna unguiculata* L. Walp), a renewed multipurpose crop for a more sustainable agri-food system: nutritional advantages and constraints. J. Sci. Food Agric. 96 (9), 2941–2951. 10.1002/jsfa.7644 26804459

[B60] Gong X. X. An Q. Le L. Q. Geng F. Jiang L. Z. Yan J. (2022). Prospects of cereal protein-derived bioactive peptides: sources, bioactivities diversity, and production. Crit. Rev. Food Sci. Nutr. 62 (11), 2855–2871. 10.1080/10408398.2020.1860897 33325758

[B61] Görgüç A. Gençdag E. Yilmaz F. M. (2020). Bioactive peptides derived from plant origin by-products: biological activities and techno-functional utilizations in food developments - a review. Food Res. Int. 136, 109504. 10.1016/j.foodres.2020.109504 32846583

[B62] Gracia S. R. Gaus K. Sewald N. (2009). Synthesis of chemically modified bioactive peptides: recent advances, challenges and developments for medicinal chemistry. Future Med. Chem. 1 (7), 1289–1310. 10.4155/fmc.09.97 21426104

[B63] Grancieri M. Martino H. S. D. de Mejia E. G. (2019). Chia seed (*Salvia hispanica* L.) as a source of proteins and bioactive peptides with health benefits: a review. Compr. Rev. Food Sci. Food Saf. 18 (2), 480–499. 10.1111/1541-4337.12423 33336944

[B64] Grasso N. Lynch N. L. Arendt E. K. O'Mahony J. A. (2022). Chickpea protein ingredients: a review of composition, functionality, and applications. Compr. Rev. Food Sci. Food Saf. 21 (1), 435–452. 10.1111/1541-4337.12878 34919328

[B65] Gravel A. Doyen A. (2023). Pulse globulins 11S and 7S: origins, purification methods, and techno-functional properties. J. Agric. Food Chem. 71 (6), 2704–2717. 10.1021/acs.jafc.2c07507 36722439

[B66] Grossmann L. Hinrichs J. Weiss J. (2020). Cultivation and downstream processing of microalgae and cyanobacteria to generate protein-based technofunctional food ingredients. Crit. Rev. Food Sci. Nutr. 60 (17), 2961–2989. 10.1080/10408398.2019.1672137 31595777

[B67] Gu M. Chen H. P. Zhao M. M. Wang X. Yang B. Ren J. Y. (2015). Identification of antioxidant peptides released from defatted walnut (*Juglans Sigillata Dode*) meal proteins with pancreatin. Lwt-Food Sci. Technol. 60 (1), 213–220. 10.1016/j.lwt.2014.07.052

[B68] Gulati P. Brahma S. Rose D. J. (2020). “Impacts of extrusion processing on nutritional components in cereals and legumes: carbohydrates, proteins, lipids, vitamins, and minerals,” in Extrusion cooking (Elsevier), 415–443.

[B69] Guo T.-t. Wan C.-y. Huang F.-h. (2019). Preparation and bioactivity evaluation of low salt peptide from sunflower seed meal.

[B70] Guo X. N. Zhu K. X. Zhang H. Yao H. Y. (2010). Anti-tumor activity of a novel protein obtained from tartary buckwheat. Int. J. Mol. Sci. 11 (12), 5201–5211. 10.3390/ijms11125201 21614202 PMC3100852

[B71] Gupta N. Srivastava N. Bhagyawant S. S. (2018). Vicilin-A major storage protein of mungbean exhibits antioxidative potential, antiproliferative effects and ACE inhibitory activity. Plos One 13 (2), e0191265. 10.1371/journal.pone.0191265 29408872 PMC5800569

[B72] Hao Y. Q. Fan X. Guo H. M. Yao Y. Ren G. X. Lv X. L. (2020). Overexpression of the bioactive lunasin peptide in soybean and evaluation of its anti-inflammatory and anti-cancer activities *in vitro* . J. Biosci. Bioeng. 129 (4), 395–404. 10.1016/j.jbiosc.2019.11.001 31784283

[B73] Hao Y. Q. Guo H. M. Hong Y. C. Fan X. Su Y. M. Yang X. S. (2022). Lunasin peptide promotes lysosome-mitochondrial mediated apoptosis and mitotic termination in MDA-MB-231 cells. Food Sci. Hum. Wellness 11 (6), 1598–1606. 10.1016/j.fshw.2022.06.018

[B74] Hassan B. Akcakanat A. Holder A. M. Meric-Bernstam F. (2013). Targeting the PI3-Kinase/Akt/mTOR signaling pathway. Surg. Oncol. Clin. N. Am. 22(4), 641. 664. 10.1016/j.soc.2013.06.008 24012393 PMC3811932

[B75] Helton E. S. Chen X. B. (2007). P53 modulation of the DNA damage response. J. Cell. Biochem. 100 (4), 883–896. 10.1002/jcb.21091 17031865

[B76] Hernandez D. F. Mojica L. de Mejia E. G. (2024). Legume-derived bioactive peptides: role in cardiovascular disease prevention and control. Curr. Opin. Food Sci. 56, 101132. 10.1016/j.cofs.2024.101132

[B77] Hernández-Ledesma B. Hsieh C. C. (2017). Chemopreventive role of food-derived proteins and peptides: a review. Crit. Rev. Food Sci. Nutr. 57 (11), 2358–2376. 10.1080/10408398.2015.1057632 26565142

[B78] Hernández-Ledesma B. Hsieh C. C. de Lumen B. O. (2009). Lunasin, a novel seed peptide for cancer prevention. Peptides 30 (2), 426–430. 10.1016/j.peptides.2008.11.002 19056440

[B79] Hernández-Ledesma B. Hsieh C. C. de Lumen B. O. (2011). Relationship between lunasin's sequence and its inhibitory activity of histones H3 and H4 acetylation. Mol. Nutr. and Food Res. 55 (7), 989–998. 10.1002/mnfr.201000632 21618425

[B80] Horner K. Drummond E. Brennan L. (2016). Bioavailability of milk protein-derived bioactive peptides: a glycaemic management perspective. Nutr. Res. Rev. 29 (1), 91–101. 10.1017/s0954422416000032 27109024

[B81] Hossain Z. Johnson E. N. Wang L. Blackshaw R. E. Gan Y. T. (2019). Comparative analysis of oil and protein content and seed yield of five *Brassicaceae* oilseeds on the Canadian prairie. Industrial Crops Prod. 136, 77–86. 10.1016/j.indcrop.2019.05.001

[B82] Hsieh C. C. Hernández-Ledesma B. de Lumen B. (2011). Cell proliferation inhibitory and apoptosis-inducing properties of anacardic acid and lunasin in human breast cancer MDA-MB-231 cells. Food Chem. 125 (2), 630–636. 10.1016/j.foodchem.2010.09.051

[B83] Hsieh C. C. Wang C. H. Huang Y. S. (2016). Lunasin attenuates obesity-associated metastasis of 4T1 breast cancer cell through anti-inflammatory property. Int. J. Mol. Sci. 17 (12), 2109. 10.3390/ijms17122109 27983683 PMC5187909

[B84] Hsieh C. C. Wu C. H. Peng S. H. Chang C. H. (2023). Seed-derived peptide lunasin suppressed breast cancer cell growth by regulating inflammatory mediators, aromatase, and estrogen receptors. Food and Nutr. Res. 67. 10.29219/fnr.v67.8991 PMC9899045 36794014

[B85] Huang J. (2021). Current developments of targeting the p53 signaling pathway for cancer treatment. Pharmacol. and Ther. 220, 107720. 10.1016/j.pharmthera.2020.107720 33130194 PMC7969395

[B86] Hühmer A. F. Aced G. I. Perkins M. D. Gürsoy R. N. Jois D. S. Larive C. (1997). Separation and analysis of peptides and proteins. Anal. Chem. 69 (12), 29–58. 10.1021/a1970003s 9195854

[B87] Hwang J. Yadav D. Lee P. C. W. Jin J. O. (2022). Immunomodulatory effects of polysaccharides from marine algae for treating cancer, infectious disease, and inflammation. Phytotherapy Res. 36 (2), 761–777. 10.1002/ptr.7348 34962325

[B88] Ikeda K. Kitamura A. Machida H. Watanabe M. Negishi H. Hiraoka J. (2003). Effect of *Undaria pinnatifida* (Wakame) on the development of cerebrovascular diseases in stroke-prone spontaneously hypertensive rats. Clin. Exp. Pharmacol. Physiology 30 (1-2), 44–48. 10.1046/j.1440-1681.2003.03786.x 12542452

[B89] Ilhan-Ayisigi E. Budak G. Celiktas M. S. Sevimli-Gur C. Yesil-Celiktas O. (2021). Anticancer activities of bioactive peptides derived from rice husk both in free and encapsulated form in chitosan. J. Industrial Eng. Chem. 103, 381–391. 10.1016/j.jiec.2021.08.006

[B90] Indiano-Romacho P. Fernández-Tomé S. Amigo L. Hernández-Ledesma B. (2019). Multifunctionality of lunasin and peptides released during its simulated gastrointestinal digestion. Food Res. Int. 125, 108513. 10.1016/j.foodres.2019.108513 31554062

[B91] Inthuwanarud K. Sangvanich P. Puthong S. Karnchanatat A. (2016). Antioxidant and antiproliferative activities of protein hydrolysate from the rhizomes of Zingiberaceae plants. Pak. J. Pharm. Sci. 29 (6), 1893–1900. 28375103

[B92] Jaeger A. Zannini E. Sahin A. W. Arendt E. K. (2021). Barley protein properties, extraction and applications, with a focus on brewers' spent grain protein. Foods 10 (6), 1389. 10.3390/foods10061389 34208463 PMC8234785

[B93] Jahanbani R. Ghaffari S. M. Salami M. Vahdati K. Sepehri H. Sarvestani N. N. (2016). Antioxidant and anticancer activities of walnut (*Juglans regia* L.) protein hydrolysates using different proteases. Plant Foods Hum. Nutr. 71 (4), 402–409. 10.1007/s11130-016-0576-z 27679440 PMC5223242

[B94] Jaradat D. M. M. (2018). Thirteen decades of peptide synthesis: key developments in solid phase peptide synthesis and amide bond formation utilized in peptide ligation. Amino Acids 50 (1), 39–68. 10.1007/s00726-017-2516-0 29185032

[B95] Jarpa-Parra M. (2018). Lentil protein: a review of functional properties and food application. An overview of lentil protein functionality. Int. J. Food Sci. Technol. 53 (4), 892–903. 10.1111/ijfs.13685

[B96] Jeong H. J. Jeong J. B. Hsieh C. C. Hernández-Ledesma B. de Lumen B. (2010). Lunasin is prevalent in barley and is bioavailable and bioactive in *in vivo* and *in vitro* studies. Nutr. Cancer-an Int. J. 62 (8), 1113–1119. 10.1080/01635581.2010.515529 21058199

[B97] Jeong H. J. Lam Y. de Lumen B. O. (2002). Barley lunasin suppresses *ras*-induced colony formation and inhibits core histone acetylation in mammalian cells. J. Agric. Food Chem. 50 (21), 5903–5908. 10.1021/jf0256945 12358457

[B98] Jia L. T. Wang L. Liu C. Liang Y. Lin Q. L. (2021). Bioactive peptides from foods: production, function, and application. Food and Funct. 12 (16), 7108–7125. 10.1039/d1fo01265g 34223585

[B99] Jiang N. N. Dai Q. J. Su X. R. Fu J. J. Feng X. C. Peng J. (2020). Role of PI3K/AKT pathway in cancer: the framework of malignant behavior. Mol. Biol. Rep. 47 (6), 4587–4629. 10.1007/s11033-020-05435-1 32333246 PMC7295848

[B100] Jiang Q. Q. Pan Y. Cheng Y. P. Li H. L. Liu D. D. Li H. (2016). Lunasin suppresses the migration and invasion of breast cancer cells by inhibiting matrix metalloproteinase-2/-9 via the FAK/Akt/ERK and NF-κB signaling pathways. Oncol. Rep. 36 (1), 253–262. 10.3892/or.2016.4798 27175819

[B101] Johansson S. Gullbo J. Lindholm P. Ek B. Thunberg E. Samuelsson G. (2003). Small, novel proteins from the mistletoe *Phoradendron tomentosum* exhibit highly, selective cytotoxicity to human breast cancer cells. Cell. Mol. Life Sci. 60 (1), 165–175. 10.1007/s000180300011 12613665 PMC11146089

[B102] Johnson L. A. Suleiman T. M. Lusas E. W. (1979). Sesame protein: a review and prospectus. J. Am. Oil Chem. Soc. 56 (3), 463–468. 10.1007/bf02671542 395182

[B103] Juárez-Chairez M. F. Cid-Gallegos M. S. Meza-Márquez O. G. Jiménez-Martínez C. (2022). Biological functions of peptides from legumes in gastrointestinal health. A review legume peptides with gastrointestinal protection. J. Food Biochem. 46 (10), e14308. 10.1111/jfbc.14308 35770807

[B104] Jung S. Rickert D. A. Deak N. A. Aldin E. D. Recknor J. Johnson L. A. (2003). Comparison of Kjeldahl and Dumas methods for determining protein contents of soybean products. J. Am. Oil Chem. Soc. 80 (12), 1169–1173. 10.1007/s11746-003-0837-3

[B105] Kang H. K. Lee H. H. Seo C. H. Park Y. (2019). Antimicrobial and immunomodulatory properties and applications of marine-derived proteins and peptides. Mar. Drugs 17 (6), 350. 10.3390/md17060350 31212723 PMC6628016

[B106] Kang H. K. Seo C. H. Park Y. (2015). Marine peptides and their anti-infective activities. Mar. Drugs 13 (1), 618–654. 10.3390/md13010618 25603351 PMC4306955

[B107] Kannan A. Hettiarachchy N. S. Lay J. O. Liyanage R. (2010). Human cancer cell proliferation inhibition by a pentapeptide isolated and characterized from rice bran. Peptides 31 (9), 1629–1634. 10.1016/j.peptides.2010.05.018 20594954

[B108] Khosravi R. Maya R. Gottlieb T. Oren M. Shiloh Y. Shkedy D. (1999). Rapid ATM-dependent phosphorylation of MDM2 precedes p53 accumulation in response to DNA damage. Proc. Natl. Acad. Sci. U. S. A. 96 (26), 14973–14977. 10.1073/pnas.96.26.14973 10611322 PMC24757

[B109] Khurshid Y. Syed B. Simjee S. U. Beg O. Ahmed A. (2020). Antiproliferative and apoptotic effects of proteins from black seeds (*Nigella sativa*) on human breast MCF-7 cancer cell line. Bmc Complementary Med. Ther. 20 (1), 5. 10.1186/s12906-019-2804-1 PMC7076859 32020890

[B110] Klupšaitė D. Juodeikienė G. J. C. T. (2015). Legume: composition, protein extraction and functional properties. A review. A Rev. 66 (1), 5–12. 10.5755/j01.ct.66.1.12355

[B111] Kuerban A. Al-Malki A. L. Kumosani T. A. Sheikh R. A. Al-Abbasi F. A. M. Alshubaily F. A. (2020). Identification, protein antiglycation, antioxidant, antiproliferative, and molecular docking of novel bioactive peptides produced from hydrolysis ofLens culinaris. J. Food Biochem. 44 (12), e13494. 10.1111/jfbc.13494 33015836

[B112] Kumar M. Suhag R. Hasan M. Dhumal S. Pandiselvam R. Senapathy M. (2023). Black soybean (<i>Glycine max</i> (L.) Merr.): paving the way toward new nutraceutical. Crit. Rev. Food Sci. Nutr. 63 (23), 6208–6234. 10.1080/10408398.2022.2029825 35139704

[B113] Kumar S. Pandey G. (2020). Biofortification of pulses and legumes to enhance nutrition. Heliyon 6 (3), e03682. 10.1016/j.heliyon.2020.e03682 32258500 PMC7114740

[B114] Lam S. K. Ng T. B. (2011). Apoptosis of human breast cancer cells induced by hemagglutinin from *Phaseolus vulgaris* cv. Legumi secchi. Food Chem. 126 (2), 595–602. 10.1016/j.foodchem.2010.11.049

[B115] Larsen P. O. (1980). “Physical and chemical properties of amino acids,” in Amino acids and derivatives (Elsevier), 225–269.

[B116] Lawrenson S. B. (2018). Greener solvents for solid-phase organic synthesis. Pure Appl. Chem. 90 (1), 157–165. 10.1515/pac-2017-0505

[B117] Lee J. H. Yang S. B. Lee J. H. Lim H. Lee S. Kang T. B. (2023). Doxorubicin covalently conjugated heparin displays anti-cancer activity as a self-assembled nanoparticle with a low-anticoagulant effect. Carbohydr. Polym. 314, 120930. 10.1016/j.carbpol.2023.120930 37173028

[B118] Li L. L. Wang Y. T. Yuan J. Y. Liu Z. Y. Ye C. Q. Qin S. (2020). *Undaria pinnatifida*improves obesity-related outcomes in association with gut microbiota and metabolomics modulation in high-fat diet-fed mice. Appl. Microbiol. Biotechnol. 104 (23), 10217–10231. 10.1007/s00253-020-10954-9 33074417

[B119] Li R. Hettiarachchy N. Mahadevan M. (2014a). Rice bran derived pentapeptide-induced apoptosis in human breast cancer cell models (MCF-7 and MDA-MB-231). Int. J. Biomed. Res. 5, 599. 10.7439/ijbr.v5i10.513

[B120] Li R. Hettiarachchy N. Mahadevan M. Sciences H. (2014b). Apoptotic pathways in human breast cancer cell models (MCF-7 and MDA-MB-231) induced by rice bran derived pentapeptide. 4(5)**,** 13–21.

[B121] Li Z. R. Jia R. B. Luo D. H. Lin L. Z. Zheng Q. W. Zhao M. M. (2021). The positive effects and underlying mechanisms of *Undaria pinnatifida* polysaccharides on type 2 diabetes mellitus in rats. Food and Funct. 12 (23), 11898–11912. 10.1039/d1fo01838h 34739010

[B122] Liang Y. J. Liu J. Feng Z. H. (2013). The regulation of cellular metabolism by tumor suppressor p53. Cell. Biosci. 3, 9. 10.1186/2045-3701-3-9 23388203 PMC3573943

[B123] Liao W. Z. Lai T. Chen L. Y. Fu J. N. Sreenivasan S. T. Yu Z. Q. (2016). Synthesis and characterization of a walnut peptides-zinc complex and its antiproliferative activity against human breast carcinoma cells through the induction of apoptosis. J. Agric. Food Chem. 64 (7), 1509–1519. 10.1021/acs.jafc.5b04924 26878665

[B124] Liao Y. Hung M. C. (2010). Physiological regulation of Akt activity and stability. Am. J. Transl. Res. 2 (1), 19–42. 20182580 PMC2826820

[B125] Lin H. X. Tan B. P. Ray G. W. Zeng M. Li M. Chi S. Y. (2022). A challenge to conventional fish meal: effects of soy protein peptides on growth, histomorphology, lipid metabolism and intestinal health for juvenile pompano Trachinotus ovatus. Front. Mar. Sci. 8. 10.3389/fmars.2021.815323

[B126] Lin X. P. Wang Q. Du S. Guan Y. C. Qiu J. M. Chen X. J. (2023). Nanoparticles for co-delivery of paclitaxel and curcumin to overcome chemoresistance against breast cancer. J. Drug Deliv. Sci. Technol. 79, 104050. 10.1016/j.jddst.2022.104050

[B127] Liu J. B. Zhang D. J. Zhu Y. S. Wang Y. Z. He S. C. Zhang T. (2018). Enhancing the *in vitro* Antioxidant Capacities via the interaction of amino acids. Emir. J. Food Agric. 30 (3), 224–231. 10.9755/ejfa.2018.v30.i3.1641

[B128] Liu L. Li S. S. Zheng J. X. Bu T. T. He G. Q. Wu J. P. (2020). Safety considerations on food protein-derived bioactive peptides. Trends Food Sci. and Technol. 96, 199–207. 10.1016/j.tifs.2019.12.022

[B129] Lo B. Kasapis S. Farahnaky A. (2021). Lupin protein: isolation and techno-functional properties, a review. Food Hydrocoll. 112, 106318. 10.1016/j.foodhyd.2020.106318

[B130] Lopez J. Al-Lihaibi S. S. Alarif W. M. Abdel-Lateff A. Nogata Y. Washio K. (2016). Wewakazole B, a cytotoxic cyanobactin from the Cyanobacterium moorea producens collected in the red sea. J. Nat. Prod. 79 (4), 1213–1218. 10.1021/acs.jnatprod.6b00051 26980238

[B131] López-Barrios L. Gutiérrez-Uribe J. A. Serna-Saldívar S. O. (2014). Bioactive peptides and hydrolysates from pulses and their potential use as functional ingredients. J. Food Sci. 79 (3), R273–R283. 10.1111/1750-3841.12365 24547749

[B132] Luna-Vital D. A. Liang K. de Mejía E. G. Loarca-Piña G. (2016). Dietary peptides from the non-digestible fraction of *Phaseolus vulgaris* L. decrease angiotensin II-dependent proliferation in HCT116 human colorectal cancer cells through the blockade of the renin-angiotensin system. Food and Funct. 7 (5), 2409–2419. 10.1039/c6fo00093b 27156533

[B133] Luo W. Bai L. Y. Zhang J. Li Z. W. Liu Y. N. Tang X. Y. (2023). Polysaccharides-based nanocarriers enhance the anti-inflammatory effect of curcumin. Carbohydr. Polym. 311, 120718. 10.1016/j.carbpol.2023.120718 37028867

[B134] Lützkendorf J. Wieduwild E. Nerger K. Lambrecht N. Schmoll H.-J. Müller-Tidow C. (2017). Resistance for genotoxic damage in mesenchymal stromal cells is increased by hypoxia but not generally dependent on p53-regulated cell cycle arrest. PLoS One 12 (1), e0169921. 10.1371/journal.pone.0169921 28081228 PMC5231334

[B135] Lv L. L. Zhao B. G. Kang J. Li S. J. Wu H. J. (2023). Trend of disease burden and risk factors of breast cancer in developing countries and territories, from 1990 to 2019: results from the Global Burden of Disease Study 2019. Front. Public Health 10, 1078191. 10.3389/fpubh.2022.1078191 36726635 PMC9884979

[B136] Ma S. H. Huang D. Zhai M. X. Yang L. B. Peng S. Chen C. X. (2015). Isolation of a novel bio-peptide from walnut residual protein inducing apoptosis and autophagy on cancer cells. Bmc Complementary Altern. Med. 15, 413. 10.1186/s12906-015-0940-9 PMC4656182 26593407

[B137] Majumdar S. Siahaan T. J. (2012). Peptide-mediated targeted drug delivery. Med. Res. Rev. 32 (3), 637–658. 10.1002/med.20225 20814957

[B138] Mao X. L. Bai L. Fan X. D. Zhang X. W. (2017). Anti-proliferation peptides from protein hydrolysates of Pyropia haitanensis. J. Appl. Phycol. 29 (3), 1623–1633. 10.1007/s10811-016-1037-7

[B139] Marcela G. M. Eva R. G. del Carmen R. R. M. Rosalva M. E. (2016). Evaluation of the antioxidant and antiproliferative effects of three peptide fractions of germinated soybeans on breast and cervical cancer cell lines. Plant Foods Hum. Nutr. 71 (4), 368–374. 10.1007/s11130-016-0568-z 27401682

[B140] Marei H. E. Althani A. Afifi N. Hasan A. Caceci T. Pozzoli G. (2021). p53 signaling in cancer progression and therapy. Cancer Cell. Int. 21 (1), 703. 10.1186/s12935-021-02396-8 34952583 PMC8709944

[B141] Massantini R. Frangipane M. T. (2022). Progress in almond quality and sensory assessment: an overview. Agriculture-Basel 12 (5), 710. 10.3390/agriculture12050710

[B142] Masui H. Fuse S. (2022). Recent advances in the solid- and solution-phase synthesis of peptides and proteins using microflow technology. Org. Process Res. and Dev. 26 (6), 1751–1765. 10.1021/acs.oprd.2c00074

[B143] Matheny R. W. Adamo M. L. (2009). Current perspectives on Akt akt-ivation and akt-ions. Exp. Biol. Med. 234 (11), 1264–1270. 10.3181/0904-mr-138 19596822

[B144] Matheu A. Maraver A. Serrano M. J. C. r. (2008). The Arf/p53 pathway in cancer and aging. Cancer Res. 68 (15), 6031–6034. 10.1158/0008-5472.CAN-07-6851 18676821

[B145] Miner-Williams W. M. Stevens B. R. Moughan P. J. (2014). Are intact peptides absorbed from the healthy gut in the adult human? Nutr. Res. Rev. 27 (2), 308–329. 10.1017/s0954422414000225 25623084

[B146] Miricescu D. Diaconu C. C. Stefani C. Stanescu A. M. A. Totan A. Rusu I. R. (2020). The serine/threonine protein kinase (Akt)/Protein kinase B (PkB) signaling pathway in breast cancer. J. Mind Med. Sci. 7 (1), 34–39. 10.22543/7674.71.P3439

[B147] Mirzaei M. Mirdamadi S. Safavi M. Soleymanzadeh N. (2020). The stability of antioxidant and ACE-inhibitory peptides as influenced by peptide sequences. Lwt-Food Sci. Technol. 130, 109710. 10.1016/j.lwt.2020.109710

[B148] Montet X. Funovics M. Montet-Abou K. Weissleder R. Josephson L. (2006). Multivalent effects of RGD peptides obtained by nanoparticle display. J. Med. Chem. 49 (20), 6087–6093. 10.1021/jm060515m 17004722

[B149] Morgensztern D. McLeod H. (2005). PI3K/Akt/mTOR pathway as a target for cancer therapy. Anticancer. Drugs 16 (8), 797–803. 10.1097/01.cad.0000173476.67239.3b 16096426

[B150] Naik A. Raghavendra S. N. Raghavarao K. (2012). Production of coconut protein powder from coconut wet processing waste and its characterization. Appl. Biochem. Biotechnol. 167 (5), 1290–1302. 10.1007/s12010-012-9632-9 22434355

[B151] Nair R. M. Yang R. Y. Easdown W. J. Thavarajah D. Thavarajah P. Hughes J. D. (2013). Biofortification of mungbean (Vigna radiata) as a whole food to enhance human health. J. Sci. Food Agric. 93 (8), 1805–1813. 10.1002/jsfa.6110 23426879

[B152] Naman C. B. Rattan R. Nikoulina S. E. Lee J. Miller B. W. Moss N. A. (2017). Integrating molecular networking and biological assays to target the isolation of a cytotoxic cyclic octapeptide, samoamide A, from an American Samoan marine Cyanobacterium. J. Nat. Prod. 80 (3), 625–633. 10.1021/acs.jnatprod.6b00907 28055219 PMC5758054

[B153] Németh R. Tömösközi S. (2021). Rye: current state and future trends in research and applications. Acta Aliment. 50 (4), 620–640. 10.1556/066.2021.00162

[B154] Noorolyai S. Shajari N. Baghbani E. Sadreddini S. Baradaran B. (2019). The relation between PI3K/AKT signalling pathway and cancer. Gene 698, 120–128. 10.1016/j.gene.2019.02.076 30849534

[B155] Nwachukwu I. D. Aluko R. E. J. J. o.F. B. (2019) Anticancer and antiproliferative properties of food-derived protein hydrolysates and peptides, 7.

[B156] Östbring K. Tullberg C. Burri S. Malmqvist E. Rayner M. (2019). Protein recovery from rapeseed press cake: varietal and processing condition effects on yield, emulsifying capacity and antioxidant activity of the protein rich extract. Foods 8 (12), 627. 10.3390/foods8120627 31805678 PMC6963604

[B157] Pagels F. Guedes A. C. Amaro H. M. Kijjoa A. Vasconcelos V. (2019). Phycobiliproteins from cyanobacteria: chemistry and biotechnological applications. Biotechnol. Adv. 37 (3), 422–443. 10.1016/j.biotechadv.2019.02.010 30797095

[B158] Pardeshi S. R. Deshmukh N. S. Telange D. R. Nangare S. N. Sonar Y. Y. Lakade S. H. (2023). Process development and quality attributes for the freeze-drying process in pharmaceuticals, biopharmaceuticals and nanomedicine delivery: a state-of-the-art review. Future J. Pharm. Sci. 9 (1), 99. 10.1186/s43094-023-00551-8

[B159] Park S. J. Ryu J. Kim I. H. Choi Y. H. Nam T. J. (2014). Induction of apoptosis by a peptide from Porphyra yezoensis: regulation of the insulin-like growth factor I receptor signaling pathway in MCF-7 cells. Int. J. Oncol. 45 (3), 1011–1016. 10.3892/ijo.2014.2509 24970277 PMC4121416

[B160] Pei J. Y. Gao X. C. Pan D. D. Hua Y. He J. Liu Z. (2022). Advances in the stability challenges of bioactive peptides and improvement strategies. Curr. Res. Food Sci. 5, 2162–2170. 10.1016/j.crfs.2022.10.031 36387592 PMC9664347

[B161] Peng D. F. Ye J. T. Jin W. P. Yang J. Geng F. Deng Q. C. (2022). A review on the utilization of flaxseed protein as interfacial stabilizers for food applications. J. Am. Oil Chem. Soc. 99 (9), 723–737. 10.1002/aocs.12621

[B162] Pennington M. W. Zell B. Bai C. (2021) Commercial manufacturing of current good manufacturing practice peptides spanning the gamut from neoantigen to commercial large-scale products, 9.100071

[B163] Prados I. M. Orellana J. M. Marina M. L. García M. C. (2020). Identification of peptides potentially responsible for *in vivo* hypolipidemic activity of a hydrolysate from olive seeds. J. Agric. Food Chem. 68 (14), 4237–4244. 10.1021/acs.jafc.0c01280 32186189

[B164] Punia S. Dhull S. B. Sandhu K. S. Kaur M. Purewal S. S. J. L. S. (2020). Kidney bean (Phaseolus vulgaris) starch: a review. A Rev. 2 (3), e52. 10.1002/leg3.52

[B165] Quintal-Bojórquez N. D. Carrillo-Cocom L. M. Hernández-Alvarez A. J. Segura-Campos M. R. (2021). Anticancer activity of protein fractions from chia (Salvia hispanica L.). J. Food Sci. 86 (7), 2861–2871. 10.1111/1750-3841.15780 34076264

[B166] Rahman M. F. A. Elhawary E. Hafez A. M. Capanoglu E. Fang Y. J. Farag M. A. (2024). How does olive seed chemistry, health benefits and action mechanisms compare to its fruit oil? A comprehensive review for valorization purposes and maximizing its health benefits. Food Biosci. 59, 104017. 10.1016/j.fbio.2024.104017

[B167] Ramkisson S. Dwarka D. Venter S. Mellem J. J. (2020). *In vitro* anticancer and antioxidant potential of Amaranthus cruentus protein and its hydrolysates. Food Sci. Technol. 40, 634–639. 10.1590/fst.36219

[B168] Ramsey J. D. Flynn N. H. (2015). Cell-penetrating peptides transport therapeutics into cells. Pharmacol. and Ther. 154, 78–86. 10.1016/j.pharmthera.2015.07.003 26210404

[B169] Rascio F. Spadaccino F. Rocchetti M. T. Castellano G. Stallone G. Netti G. S. (2021). The pathogenic role of PI3K/AKT pathway in cancer onset and drug resistance: an updated review. Cancers 13 (16), 3949. 10.3390/cancers13163949 34439105 PMC8394096

[B170] Rayaprolu S. J. Hettiarachchy N. S. Horax R. Phillips G. K. Mahendran M. Chen P. Y. (2017). Soybean peptide fractions inhibit human blood, breast and prostate cancer cell proliferation. J. Food Sci. Technology-Mysore 54 (1), 38–44. 10.1007/s13197-016-2426-2 PMC5305699 28242901

[B171] Ren J. Li S. J. Song C. L. Sun X. H. Liu X. L. (2021). Black soybean-derived peptides exerted protective effect against alcohol-induced liver injury in mice. J. Funct. Foods 87, 104828. 10.1016/j.jff.2021.104828

[B172] Ricci C. Pastukh V. Leonard J. Turrens J. Wilson G. Schaffer D. (2008). Mitochondrial DNA damage triggers mitochondrial-superoxide generation and apoptosis. Am. J. Physiology-Cell Physiology 294 (2), C413–C422. 10.1152/ajpcell.00362.2007 18077603

[B173] Saranraj P. Sivasakthi S. (2014). Spirulina platensis–food for future: a review. 4(1)**,** 26–33.

[B174] Segura-Campos M. Chel-Guerrero L. Betancur-Ancona D. Hernandez-Escalante V. (2011). Bioavailability of bioactive peptides. 27(3)**,** 213–226. 10.1080/87559129.2011.563395

[B175] Shah N. K. Guzmán E. A. T. Wang Z. Meenach S. A. (2020). “Routes of administration for nanocarriers,” in Nanoparticles for biomedical applications (Elsevier), 67–87.

[B176] Shanthakumar P. Klepacka J. Bains A. Chawla P. Dhull S. B. Najda A. (2022). The current situation of pea protein and its application in the food industry. Molecules 27 (16), 5354. 10.3390/molecules27165354 36014591 PMC9412838

[B177] Shewry P. R. (2007). Improving the protein content and composition of cereal grain. J. Cereal Sci. 46 (3), 239–250. 10.1016/j.jcs.2007.06.006

[B178] Shi H. Y. Ji H. F. Zhu F. Z. Zhao Y. Yin Y. Y. Shu F. J. (2022). Antitumor potential of peptides isolated from Brucea javanica globulin fraction on MCF-7 cells. Pharmacogn. Mag. 18 (80), 1129–1136. 10.4103/pm.pm_540_21

[B179] Singh B. P. Vij S. (2018). *In vitro* stability of bioactive peptides derived from fermented soy milk against heat treatment, pH and gastrointestinal enzymes. Lwt-Food Sci. Technol. 91, 303–307. 10.1016/j.lwt.2018.01.066

[B180] Sontakke S. B. Jung J. H. Piao Z. Chung H. J. (2016). Orally available collagen tripeptide: enzymatic stability, intestinal permeability, and absorption of gly-pro-hyp and pro-hyp. J. Agric. Food Chem. 64 (38), 7127–7133. 10.1021/acs.jafc.6b02955 27573716

[B181] Spaen J. Silva J. V. C. (2021). Oat proteins: review of extraction methods and techno-functionality for liquid and semi-solid applications. Lwt-Food Sci. Technol. 147, 111478. 10.1016/j.lwt.2021.111478

[B182] Stalmans S. Wynendaele E. Bracke N. Gevaert B. D'Hondt M. Peremans K. (2013). Chemical-functional diversity in cell-penetrating peptides. Plos One 8 (8), e71752. 10.1371/journal.pone.0071752 23951237 PMC3739727

[B183] Sudheesh C. Bhat Z. R. Aaliya B. Sunooj K. V. (2022). “Cereal proteins,” in Nutraceuticals and health care (Elsevier), 29–60.

[B184] Sun B. Ross S. M. Rowley S. Adeleye Y. Clewell R. A. (2017). Contribution of ATM and ATR kinase pathways to p53-mediated response in etoposide and methyl methanesulfonate induced DNA damage. Environ. Mol. Mutagen. 58 (2), 72–83. 10.1002/em.22070 28195382

[B185] Suo Q. S. Yue Y. Wang J. Wu N. Geng L. H. Zhang Q. B. (2022). Isolation, identification and *in vivo* antihypertensive effect of novel angiotensin I-converting enzyme (ACE) inhibitory peptides from *Spirulina* protein hydrolysate. Food and Funct. 13 (17), 9108–9118. 10.1039/d2fo01207c 35946851

[B186] Taghizadeh M. S. Niazi A. Moghadam A. Afsharifar A. R. (2020). The potential application of the protein hydrolysates of three medicinal plants: cytotoxicity and functional properties. J. Food Sci. 85 (10), 3160–3167. 10.1111/1750-3841.15379 32885425

[B187] Tan M. Nawaz M. A. Buckow R. (2023). Functional and food application of plant proteins - a review. Food Rev. Int. 39 (5), 2428–2456. 10.1080/87559129.2021.1955918

[B188] Taniya M. S. Reshma M. V. Shanimol P. S. Krishnan G. Priya S. (2020). Bioactive peptides from amaranth seed protein hydrolysates induced apoptosis and antimigratory effects in breast cancer cells. Food Biosci. 35, 100588. 10.1016/j.fbio.2020.100588

[B189] Temba M. C. Njobeh P. B. Adebo O. A. Olugbile A. O. Kayitesi E. (2016). The role of compositing cereals with legumes to alleviate protein energy malnutrition in Africa. Int. J. Food Sci. Technol. 51 (3), 543–554. 10.1111/ijfs.13035

[B190] Tewari D. Patni P. Bishayee A. Sah A. N. Bishayee A. (2022). Natural products targeting the PI3K-Akt-mTOR signaling pathway in cancer: a novel therapeutic strategy. Seminars Cancer Biol. 80, 1–17. 10.1016/j.semcancer.2019.12.008 31866476

[B191] Tok K. Moulahoum H. Kocazorbaz E. K. Zihnioglu F. (2021). Bioactive peptides with multiple activities extracted from Barley (*Hordeum vulgare* L.) grain protein hydrolysates: biochemical analysis and computational identification. J. Food Process. Preserv. 45 (1). 10.1111/jfpp.15024

[B192] Toomer O. T. (2018). Nutritional chemistry of the peanut (*Arachis hypogaea*). Crit. Rev. Food Sci. Nutr. 58 (17), 3042–3053. 10.1080/10408398.2017.1339015 28662347

[B193] Trinidad Calderón P. A. (2022). Development of a novel zein-derived peptide: *in vitro* assays, and 2D-and 3D-cell models.

[B194] Trotta A. P. Chipuk J. E. (2017). Mitochondrial dynamics as regulators of cancer biology. Cell. Mol. Life Sci. 74 (11), 1999–2017. 10.1007/s00018-016-2451-3 28083595 PMC5419868

[B195] Turky N. O. Abdelmonem N. A. Tammam S. N. Gad M. Z. Breitinger H. G. Breitinger U. (2024). Antibacterial and *in vitro* anticancer activities of the antimicrobial peptide NRC-07 encapsulated in chitosan nanoparticles. J. Peptide Sci. 30 (4), e3550. 10.1002/psc.3550 37853814

[B196] Vásquez-Villanueva R. Muñoz-Moreno L. Carmena M. J. Marina M. L. García M. C. (2018). *In vitro* antitumor and hypotensive activity of peptides from olive seeds. J. Funct. Foods 42, 177–184. 10.1016/j.jff.2017.12.062

[B197] Vermeirssen V. Van Camp J. Verstraete W. (2004). Bioavailability of angiotensin I converting enzyme inhibitory peptides. Br. J. Nutr. 92 (3), 357–366. 10.1079/bjn20041189 15469639

[B198] Wang J. Wu S. G. (2023). Breast cancer: an overview of current therapeutic strategies, challenge, and perspectives. Breast Cancer-Targets Ther. 15, 721–730. 10.2147/bctt.S432526 PMC10596062 37881514

[B199] Wang J. P. Liu J. M. John A. Jiang Y. M. Zhu H. Yang B. (2022a). Structure identification of walnut peptides and evaluation of cellular antioxidant activity. Food Chem. 388, 132943. 10.1016/j.foodchem.2022.132943 35436638

[B200] Wang L. Wang N. X. Zhang W. P. Cheng X. R. Yan Z. B. Shao G. (2022b). Therapeutic peptides: current applications and future directions. Signal Transduct. Target. Ther. 7 (1), 48. 10.1038/s41392-022-00904-4 35165272 PMC8844085

[B201] Wang L. F. Zhang J. Yuan Q. Xie H. H. Shi J. Y. Ju X. R. (2016). Separation and purification of an anti-tumor peptide from rapeseed (Brassica campestris L.) and the effect on cell apoptosis. Food and Funct. 7 (5), 2239–2248. 10.1039/c6fo00042h 27116475

[B202] Wang Q. L. Xiong Y. L. L. (2019). Processing, nutrition, and functionality of hempseed protein: a review. Compr. Rev. Food Sci. Food Saf. 18 (4), 936–952. 10.1111/1541-4337.12450 33336999

[B203] Wang X. C. Liu H. Ni Y. Q. Shen P. B. Han X. Z. (2022c). Lactate shuttle: from substance exchange to regulatory mechanism. Hum. Cell. 35 (1), 1–14. 10.1007/s13577-021-00622-z 34606041

[B204] Wang Z. G. Zhang R. X. Zhang T. He C. S. He R. Ju X. R. (2018). *In situ* proapoptotic peptide-generating rapeseed protein-based nanocomplexes synergize chemotherapy for cathepsin-B overexpressing breast cancer. Acs Appl. Mater. and Interfaces 10 (48), 41056–41069. 10.1021/acsami.8b14001 30387987

[B205] Wang Z. J. Zhang X. W. (2016a). Characterization and antitumor activity of protein hydrolysates from Arthrospira platensis (Spirulina platensis) using two-step hydrolysis. J. Appl. Phycol. 28 (6), 3379–3385. 10.1007/s10811-016-0881-9

[B206] Wang Z. J. Zhang X. W. (2016b). Inhibitory effects of small molecular peptides from Spirulina (Arthrospira) platensis on cancer cell growth. Food and Funct. 7 (2), 781–788. 10.1039/c5fo01186h 26584028

[B207] Wang Z. J. Zhang X. W. (2017). Isolation and identification of anti-proliferative peptides from Spirulina platensis using three-step hydrolysis. J. Sci. Food Agric. 97 (3), 918–922. 10.1002/jsfa.7815 27218227

[B208] Wei K. Wei Y. Xu W. D. Lu F. Ma H. L. (2022). Corn peptides improved obesity-induced non-alcoholic fatty liver disease through relieving lipid metabolism, insulin resistance and oxidative stress. Food and Funct. 13 (10), 5782–5793. 10.1039/d2fo00199c 35537139

[B209] Wei Y. P. Xu J. D. Zhang L. Fu Y. K. Xu X. (2015). Development of novel small peptide ligands for antibody purification. Rsc Adv. 5 (82), 67093–67101. 10.1039/c5ra07829f

[B210] Wen C. T. Zhang J. X. Zhang H. H. Duan Y. Q. Ma H. L. (2020). Plant protein-derived antioxidant peptides: isolation, identification, mechanism of action and application in food systems: a review. Trends Food Sci. and Technol. 105, 308–322. 10.1016/j.tifs.2020.09.019

[B211] Wen C. T. Zhang Z. Y. Cao L. Y. Liu G. Y. Liang L. Liu X. F. (2023). Walnut protein: a rising source of high-quality protein and its updated comprehensive review. J. Agric. Food Chem. 71 (28), 10525–10542. 10.1021/acs.jafc.3c01620 37399339

[B212] Wen H. D. Zhang D. J. Liu J. B. Shang X. M. Liu X. T. Du Z. Y. (2022). Application of γ-cyclodextrin-lysozyme as host materials for encapsulation of curcumin: characterization, stability, and controlled release properties. J. Sci. Food Agric. 102 (13), 5925–5934. 10.1002/jsfa.11943 35437803

[B213] Wolosik K. Markowska A. (2019). Amaranthus cruentus taxonomy, botanical description, and review of its seed chemical composition. Nat. Product. Commun. 14 (5). 10.1177/1934578x19844141

[B214] Wong J. H. Ng T. B. (2005). Sesquin, a potent defensin-like antimicrobial peptide from ground beans with inhibitory activities toward tumor cells and HIV-1 reverse transcriptase. Peptides 26 (7), 1120–1126. 10.1016/j.peptides.2005.01.003 15949629

[B215] Xie H. H. Wang Y. M. Zhang J. Chen J. Y. Wu D. Wang L. F. (2015). Study of the fermentation conditions and the antiproliferative activity of rapeseed peptides by bacterial and enzymatic cooperation. Int. J. Food Sci. Technol. 50 (3), 619–625. 10.1111/ijfs.12682

[B216] Xu S. W. Shen Y. T. Xu J. W. Qi G. Y. Chen G. J. Wang W. Q. (2019). Antioxidant and anticancer effects in human hepatocarcinoma (HepG2) cells of papain-hydrolyzed sorghum kafirin hydrolysates. J. Funct. Foods 58, 374–382. 10.1016/j.jff.2019.05.016

[B217] Xue Z. H. Wen H. C. Zhai L. J. Yu Y. Q. Li Y. N. Yu W. C. (2015). Antioxidant activity and anti-proliferative effect of a bioactive peptide from chickpea (Cicer arietinum L.). Food Res. Int. 77, 75–81. 10.1016/j.foodres.2015.09.027

[B218] Yamaguchi M. Takeuchi M. Ebihara K. (1997). Inhibitory effect of peptide prepared from corn gluten meal on 7,12-dimethylbenz a anthracene-induced mammary tumor progression in rats. Nutr. Res. 17 (7), 1121–1130. 10.1016/s0271-5317(97)00083-3

[B219] Yamaguchi Y. Yamamoto K. Sato Y. Inoue S. Morinaga T. Hirano E. (2016). Combination of aspartic acid and glutamic acid inhibits tumor cell proliferation. Biomed. Research-Tokyo 37 (2), 153–159. 10.2220/biomedres.37.153 27108884

[B220] Yan A. F. Ren C. H. Chen T. Huo D. Jiang X. Sun H. Y. (2018). A novel caspase-6 from sea cucumber Holothuria leucospilota: molecular characterization, expression analysis and apoptosis detection. Fish and Shellfish Immunol. 80, 232–240. 10.1016/j.fsi.2018.06.006 29890217

[B221] Ye D. D. Sun L. J. Zou B. R. Zhang Q. Tan W. Y. Che W. K. (2018). Non-destructive prediction of protein content in wheat using NIRS. Spectrochimica Acta Part a-Molecular Biomol. Spectrosc. 189, 463–472. 10.1016/j.saa.2017.08.055 28843880

[B222] Yeap S. K. Yusof H. M. Mohamad N. E. Beh B. K. Ho W. Y. Ali N. M. (2013). *In vivo* immunomodulation and lipid peroxidation activities contributed to chemoprevention effects of fermented mung bean against breast cancer. Evidence-Based Complementary Altern. Med. 2013, 708464. 10.1155/2013/708464 PMC3654717 23710232

[B223] Yen Y. W. Lee Y. L. Yu L. Y. Li C. E. Shueng P. W. Chiu H. C. (2023). Fucoidan/chitosan layered PLGA nanoparticles with melatonin loading for inducing intestinal absorption and addressing triple-negative breast cancer progression. Int. J. Biol. Macromol. 250, 126211. 10.1016/j.ijbiomac.2023.126211 37562466

[B224] Yu X. Z. Zhu L. L. (2024). Nanoparticles for the treatment of bone metastasis in breast cancer: recent advances and challenges. Int. J. Nanomedicine 19, 1867–1886. 10.2147/ijn.S442768 38414525 PMC10898486

[B225] Yuan L. Chu Q. Wu X. Y. Yang B. Zhang W. Jin W. A. (2021). Anti-inflammatory and antioxidant activity of peptides from ethanol-soluble hydrolysates of sturgeon (Acipenser schrenckii) cartilage. Front. Nutr. 8, 689648. 10.3389/fnut.2021.689648 34179062 PMC8225940

[B226] Zhang D. J. Jiang K. Luo H. Zhao X. R. Yu P. Gan Y. M. (2024a). Replacing animal proteins with plant proteins: is this a way to improve quality and functional properties of hybrid cheeses and cheese analogs? Compr. Rev. Food Sci. Food Saf. 23 (1), e13336. 10.1111/1541-4337.13262 38284577

[B227] Zhang D. J. Ling X. L. Jiang K. Zhao X. R. Gan Y. M. (2024b). Development of low-fat Mozzarella cheeses enriched with soy or pea protein hydrolysates: composition, texture and functional properties during ageing. Int. J. Dairy Technol. 77 (1), 165–182. 10.1111/1471-0307.13021

[B228] Zhang D. J. Wang Y. Z. Xu M. L. Ding L. Zhang T. Liu J. B. (2017). Antioxidant synergetic effect between the peptides derived from the egg white pentapeptide Trp-Asn-Trp-Ala-Asp. Int. J. Peptide Res. Ther. 23 (4), 509–518. 10.1007/s10989-017-9585-5

[B229] Zhang D. J. Xiong J. Zhao X. R. Gan Y. M. (2023a). Anti-fatigue activities of ? aminobutyric acid-enriched soymilk in an acute exercise-treated mouse model via regulating AMPK/PGC-1a pathway. Food Biosci. 55, 103060. 10.1016/j.fbio.2023.103060

[B230] Zhang L. Y. Miao J. Y. Guo J. B. Liu J. Xia Z. Chen B. B. (2023b). Two novel angiotensin I-converting enzyme (ACE) inhibitory peptides from rice (oryza sativa L.) bran protein. J. Agric. Food Chem. 71, 4153–4162. 10.1021/acs.jafc.2c07270 36812450

[B231] Zhang P. Yang H. L. Shen W. Liu W. G. Chen L. Xiao C. S. (2020). Hypoxia-responsive polypeptide nanoparticles loaded with doxorubicin for breast cancer therapy. Acs Biomaterials Sci. and Eng. 6 (4), 2167–2174. 10.1021/acsbiomaterials.0c00125 33455312

[B232] Zhang X. X. Eden H. S. Chen X. Y. (2012). Peptides in cancer nanomedicine: drug carriers, targeting ligands and protease substrates. J. Control. Release 159 (1), 2–13. 10.1016/j.jconrel.2011.10.023 22056916 PMC3288222

[B233] Zhang Y. Z. Zhang J. Yan J. W. Qi X. R. Wang Y. H. Zheng Z. T. (2024c). Application of fermented Chinese herbal medicines in food and medicine field: from an antioxidant perspective. Trends Food Sci. and Technol. 148, 104410. 10.1016/j.tifs.2024.104410

[B234] Zhang Z. Q. Zhang Y. Zhang M. Y. Yu C. F. Yang P. P. Xu M. X. (2023c). Food-derived peptides as novel therapeutic strategies for NLRP3 inflammasome-related diseases: a systematic review. Crit. Rev. Food Sci. Nutr., 1–32. 10.1080/10408398.2023.2294164 38153262

[B235] Zheng J. (2012). Energy metabolism of cancer: glycolysis versus oxidative phosphorylation (Review). Oncol. Lett. 4 (6), 1151–1157. 10.3892/ol.2012.928 23226794 PMC3506713

[B236] Zheng Q. P. Qiu D. S. Liu X. J. Zhang L. Cai S. K. Zhang X. W. (2015). Antiproliferative effect of Dendrobium catenatum Lindley polypeptides against human liver, gastric and breast cancer cell lines. Food and Funct. 6 (5), 1489–1495. 10.1039/c5fo00060b 25811957

[B237] Zhu F. (2021). Buckwheat proteins and peptides: biological functions and food applications. Trends Food Sci. and Technol. 110, 155–167. 10.1016/j.tifs.2021.01.081

[B238] Zhu F. Cao J. R. Song Y. T. Yu P. F. Su E. R. (2023). Plant protein-derived active peptides: a comprehensive review. J. Agric. Food Chem. 71 (51), 20479–20499. 10.1021/acs.jafc.3c06882 38109192

